# Modeling BK Virus Infection in Renal Transplant Recipients

**DOI:** 10.3390/v17010050

**Published:** 2024-12-31

**Authors:** Nicholas Myers, Dana Droz, Bruce W. Rogers, Hien Tran, Kevin B. Flores, Cliburn Chan, Stuart J. Knechtle, Annette M. Jackson, Xunrong Luo, Eileen T. Chambers, Janice M. McCarthy

**Affiliations:** 1 Center for Research in Scientific Computation, Department of Mathematics, North Carolina State University, Raleigh, NC 27695, USA; nmyers2@ncsu.edu (N.M.); dedroz@ncsu.edu (D.D.); kbflores@ncsu.edu (K.B.F.); 2 Department of Surgery, Duke University, Durham, NC 27710, USAstuart.knechtle@duke.edu (S.J.K.); annette.m.jackson@duke.edu (A.M.J.); eileen.chambers@duke.edu (E.T.C.); 3 Duke Center for Human Systems Immunology, Duke University, Durham, NC 27701, USA; cliburn.chan@duke.edu (C.C.); janice.mccarthy@duke.edu (J.M.M.); 4 Department of Biostatistics and Bioinformatics, Duke University, Durham, NC 27710, USA; 5 Department of Medicine, Duke University, Durham, NC 27710, USA; xunrong.luo@duke.edu; 6 Department of Pediatrics, Duke University, Durham, NC 27710, USA

**Keywords:** kidney, renal, transplant, modeling, sensitivity, BKPyV, model calibration, immunosuppression

## Abstract

Kidney transplant recipients require a lifelong protocol of immunosuppressive therapy to prevent graft rejection. However, these same medications leave them susceptible to opportunistic infections. One pathogen of particular concern is human polyomavirus 1, also known as BK virus (BKPyV). This virus attacks kidney tubule epithelial cells and is a direct threat to the health of the graft. Current standard of care in BK virus-infected transplant recipients is reduction in immunosuppressant therapy, to allow the patient’s immune system to control the virus. This requires a delicate balance; immune suppression must be strong enough to prevent rejection, yet weak enough to allow viral clearance. We seek to model viral and immune dynamics with the ultimate goal of applying optimal control methods to this problem. In this paper, we begin with a previously published model and make simplifying assumptions that reduce the number of parameters from 20 to 14. We calibrate our model using newly available patient data and a detailed sensitivity analysis. Numerical results for multiple patients are given to show that the newer model reflects observed dynamics well.

## 1. Introduction

In any solid organ transplant, immunosuppression is necessary to prevent graft rejection. This often begins with induction treatment to prevent acute rejection immediately post-procedure [1]. A lifetime of maintenance therapy consisting of a combination of immunosuppressive drugs to prevent chronic allograft nephropathy and allograft loss is always necessary [1]. Unfortunately, immunosuppression leaves patients at risk for a multitude of opportunistic infections, particularly for high-prevalence, latent viral pathogens such as cytomegalovirus (CMV), Epstein–Barr virus (EBV) and BK virus (BKPyV). In renal transplant recipients, BKPyV is of great concern, as it infects and kills tubule epithelial cells [2]. BK viremia occurs in up to 30% of kidney transplant recipients and BK virus-associated nephropathy occurs in 1–10% of kidney transplant cases. Of those, about 15% will result in graft loss [2]. In fact, two of the three leading causes of organ failure in renal transplant patients are rejection and BKPyV nephropathy [3,4].

Our ultimate goal is to determine the optimal level of immunosuppression to allow immune system clearance of the virus, while preventing graft rejection. A first step is developing a mathematical model of virus and immune dynamics in which immunosuppression is a tunable parameter. Within-host modeling of viral infections has been an important tool in studying viral dynamics, beginning in the early 1990s with HIV [5], and extending to influenza [6], hepatitis C [7,8] and many others. These mechanistic models have been used to better understand interactions between virus particles, the cells they infect and the immune system and to optimize treatment protocols (see, for example, [9]). A number of mathematical and statistical modeling approaches have been proposed to assist in understanding different aspects of renal transplantation since the 1970s [10,11,12,13,14,15]. An immune response model for individual renal transplant patients with compartments that represent the allograft cells, a pathogen, immune system cells and a biomarker for health of the kidney was developed [16], but that model was fit using data from only a single patient. Others have paired different Kalman filtering techniques with a receding horizon control problem to create a data-driven optimal drug efficacy profile for immunosuppression treatment as a demonstration of their personalized medicine approach [17,18,19,20]; however, none of this work included patient data of any kind. Here, we describe a simplified model and calibrate it using patient data from a representative sample of five patients from two different datasets. One dataset is from the Duke Transplant Center Electronic Health Record (EHR), and the other is from CTOT-19, a study that was part of Clinical Trials in Organ Transplantation consortium (CTOT).

This paper aims to show the viability of an updated model of BKPyV infection in kidney transplant recipients using newly available data. This paper is organized as follows. We first describe the new model (model 2) as derived in Supplementary Materials S2 from [17]. We then describe the datasets provided by the Duke Transplant Center and National Institute of Health’s Clinical Trials in Organ Transplantation and the subset of patient data that was used for calibration of the new model [21,22]. We continue by presenting the calibration results. Next, we conduct stability analysis and sensitivity analysis to highlight the benefits of the new model. Finally, we will discuss the future direction of the project and how this model supports the goal of personalized medicine and the development of a tool to provide recommendations to clinicians for optimal immunosuppression treatments.

## 2. Methods and Data

### 2.1. Model Description

**Figure 1:**
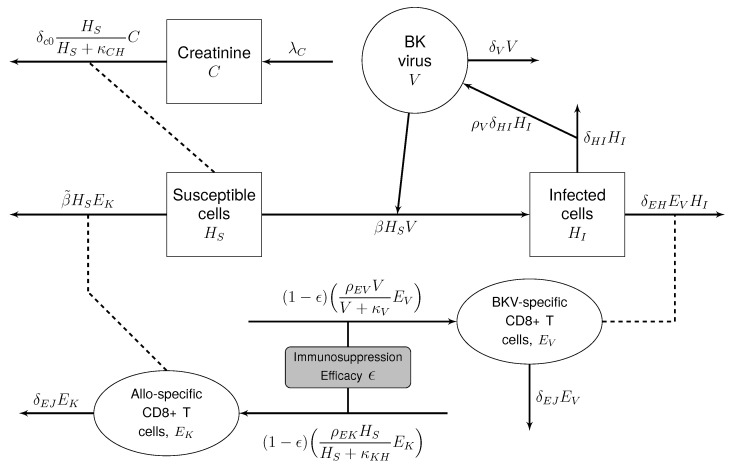
Diagram of the model 2 state interactions. There is a cyclical relationship between susceptible cells, infected cells, and BKPyV. The immunosuppression efficacy ϵ affects the growth of the immune cell states and these immune cell states affect the loss terms for their targets, susceptible cells and infected cells. Also, note that creatinine is a biomarker and does not affect the other model states.

The general form of the model has six states, as shown in Table 1. The susceptible ($H_{S}$) and infected ($H_{I}$) cells are renal tubule cells within the nephrons of the allograft, which are responsible for the removal of waste products from blood. The virus (V) infects susceptible renal tubule cells for replication, and ultimately, the cytotoxic effect of the viral infection causes damage to the allograft. While the full immune response is complex, the model focuses on the cytotoxic effect of CD8+ T cells, which are important for viral clearance and also involved in allograft rejection. We model two populations of CD8+ T cells: one set that attacks BKPyV-infected cells ($E_{V}$) and one that attacks the healthy allograft cells ($E_{K}$). We focus on T cell responses because the long-term goal is to determine individualized drug regimens that allow the elimination of BK virus while preventing organ rejection using optimal control theory. Immunosuppressants from two classes are typically lowered in response to BKPyV infection: calcineurin inhibitors and antiproliferatives. Both classes of drugs inhibit the formation and proliferation of cytotoxic T cells [23]. While other immune responses, like neutralizing antibodies and natural killer cells, are important for controlling BKPyV, we have abstracted these mechanisms in the model, and they are described generally by the viral clearance rate, $\delta_{V}$. Finally, serum creatinine levels (C) are used to assess the impairment of the kidney by BKPyV and allo-specific T cells. Creatinine is a byproduct of muscle metabolism which is then cleared by the kidney and is the primary component in the calculation of estimated glomerular filtration rate (eGFR) [24,25]. The model is given by Equations (1)–(6) with a compartmental diagram in Figure 1. The parameter descriptions and default values are found in Table 2.

The original model that was improved upon for this work was developed by one of the authors (Tran) in [17]. Changes to the model were made, leading to models 1, 1.1, and 2, as described in detail in Supplementary Materials Section S2. The differences between model 1 and model 2 occur in the expressions used to describe the immune cells in Equations (4) and (5). Model 1 includes three additional parameters and has unrealistic behavior for the CD8+ T cell compartments, which motivated the development of the model presented in this paper, model 2. Simplifying the model to fewer parameters and improving the dynamics of model states were our goals for model 2.

                        Updated Immune Response Model: Model 2

(1) $$\begin{array}{cc} {\dot{H}}_{S} & =-\beta H_{S}V-\tilde{\beta }H_{S}E_{K} \end{array}$$

(2) $$\begin{array}{cc} {\dot{H}}_{I} & =\beta H_{S}V-\delta_{HI}H_{I}-\delta_{EH}E_{V}H_{I} \end{array}$$

(3) $$\begin{array}{cc} \dot{V} & =\rho_{V}\delta_{HI}H_{I}-\delta_{V}V-\beta H_{S}V \end{array}$$

(4) $$\begin{array}{cc} {\dot{E}}_{V} & =\left(1-\epsilon \right)\left(\frac{\rho_{EV}V}{V+\kappa_{V}}E_{V}\right)-\delta_{EJ}E_{V} \end{array}$$

(5) $$\begin{array}{cc} {\dot{E}}_{K} & =\left(1-\epsilon \right)\left(\frac{\rho_{EK}H_{S}}{H_{S}+\kappa_{KH}}E_{K}\right)-\delta_{EJ}E_{K} \end{array}$$

(6) $$\begin{array}{cc} \dot{C} & =\lambda_{C}-\delta_{C0}\frac{H_{S}}{H_{S}+\kappa_{CH}}C \end{array}$$

In model 2, the susceptible cell population ($H_{S}$ in Equation (1)) has no regeneration term; therefore, the allograft loses kidney cells over time due to infection by BKPyV ($-\beta H_{S}V)$ and elimination by allograft-specific CD8+ T cells ($\tilde{\beta }H_{S}E_{K}$). These two mass action terms represent the primary threats to the survival of the allograft: BKPyV infection and organ rejection.

Viral reproduction, in Equation (3), is modeled by a mass action term describing infection ($-\beta H_{S}V$) and a linear term describing viral replication due to lysing of infected cells ($\rho_{V}\delta_{HI}H_{I}$). The constants $\rho_{V}$ and $\delta_{HI}$ represent the replication rate of virus and the lysing rate of infected cells, respectively. The remaining term in this equation, $-\delta_{V}V$, describes a constant rate of removal from the population. In addition to lysing from BK infection, infected cells are also removed at a background linear rate ($-\delta_{HI}$ in Equation (2)) and by T cell cytosis ($\delta_{EH}E_{V}H_{I}$).

Immunosuppression is modeled by a multiplicative suppression term (1−ϵ) on the production of cytotoxic T cells (Equations (4) and (5)). The parameter ϵ represents the efficacy of the immunosuppressant regimen. For example, ϵ=0.6 represents 60% efficacy in suppressing this immune effector. We assume that immunosuppression efficacy level is a constant 80%, ϵ=0.8, in this work. This relatively high choice for efficacy follows from the standard intensive drug regimen which we expect to significantly impact the production of immune cells. T cell production is modeled by a half-saturation expression (the rational terms in these equations) that increases as the threat grows or decreases as the threat subsides.

Creatinine is a product of muscle metabolism that accumulates in the blood and is eliminated by the kidney. Therefore, serum creatinine levels have been used as a biomarker for kidney function [16,19]. Creatinine is produced at a constant rate, $\lambda_{C}$, and a half-saturationfunction dependent upon cleansing rate $\delta_{C0}$ and the quantity of healthy cells ($H_{S}$) defines the removal of creatinine from the blood. Note that creatinine in the model does not effect the other states, as shown in the diagram in Figure 1.

### 2.2. Patient Data

Patient data are drawn from two cohorts. (1) We obtained patient data from over 400 kidney transplant patients from Duke Transplant Center Electronic Health Record. This includes regular measurements of BKPyV copies in the blood and serum creatinine levels. The BKPyV data come from blood work conducted both on-site at the center, which has a detection limit of 50 copies per mL, and off-site testing, with a limit of detection of 200 copies per mL, as observed in the data. (2) We downloaded from Immport [26] the data from PROTOCOL CTOT-19 [22], part of the Clinical Trials in Organ Transplantation consortium sponsored by the National Institute of Allergy and Infectious Diseases. This dataset has higher-frequency viral load measurements than other clinical trials involving kidney transplantation (e.g., CTOT-15 [21]) but extremely sparse serum creatinine data.

**Figure 2:**
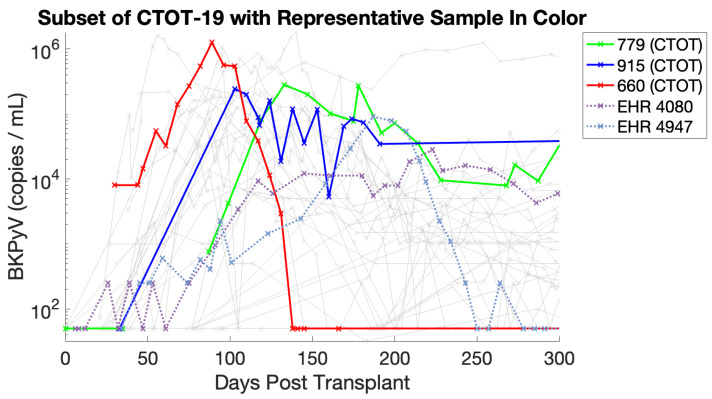
All CTOT-19 subset viral load trajectories with representative samples highlighted.

A subset of patients was drawn from the full datasets for this work. Table 3 and Table 4 show how patients were chosen for our subset. A representative sample of four patients with peak BKPyV loads above $10^{4}$ and below $10^{6}$ copies/mL occurring before 300 days post-transplant is considered, two patients from each cohort. An additional patient with higher viral load concentrations ($>10^{6}$ copies/mL) and earlier presentation (<100 days post-transplant) is chosen to further demonstrate model flexibility. Most transplant patients do not present with BKPyV; patients with a moderate peak viral load are chosen because (A) some initial virus must be present to start an infection in the model and (B) since fixed immunosuppression treatment is assumed, treatment for these patients should not change substantially, similar to a fixed treatment. We consider patients who peak before day 300 since BKPyV infection is most common within the first year and for reasonable computation times [2]. The two patients chosen from the EHR cohort are denoted #4080 and #4947, and the two patients from the CTOT-19 study are called #287779 and #287915 (anonymized IDs). The final CTOT-19 patient with a high, early peak is designated #287660. In Figure 2, see that these five patients cover the chosen subset and demonstrate the model’s viability in the group of interest.

### 2.3. Parameter Estimation

Model parameters are estimated from patient data in the EHR cohort using a two-step process. First, the viral load measurements are used to estimate the parameters directly associated with viral production and regulation: $\delta_{V},\delta_{EH},\rho_{EV},\delta_{HI},\rho_{V},\delta_{EJ},\rho_{EK}$ and the initial condition $H_{S0}$, with all other parameters fixed. Next, creatinine measurements are used to estimate the constants governing creatinine production and clearance: $\lambda_{C}$ and $\delta_{C0}$, while fixing the parameters estimated in the previous step to their estimated values. Other parameters remain fixed (see Section 3.2). Be aware that while creatinine data are sparse in the CTOT cohort, they were used to fit creatinine-related parameters.

Parameter estimates for each patient proceed by Ordinary Least Squares (OLS) [27]. To find the parameter set θ for a particular patient, we refer to the observable states of virus and creatinine as *Y* and the observation function f(x(t),t;θ). For each patient, we solve the following objective function using the *N* observations:

(7) $$\begin{array}{c} \theta_{OLS}=\arg\min_{\theta \in \Omega_{\theta }}\sum_{j=1}^{N}{\left(Y_{j}-f\left(x\left(t_{j}\right),t_{j};\theta \right)\right)}^{2}. \end{array}$$

The parameters are estimated using Matlab’s built-in solver fmincon and the Multistart feature [28]. Multistart samples 100 initial values of each parameter from their respective parameter spaces in order to make the parameter estimation process less dependent on the choice of initial guess. Table 2 gives a description of each parameter, along with its initialization value for the estimation method.

Uncertainty in the parameter estimates is carried out using the methodology presented in the work of Banks et al. (2015) [29]. Here, uncertainty analysis based on asymptotic theory is performed. For a more comprehensive analysis, subset selection is utilized to determine groups of parameters for which the asymptotic standard errors would be reasonable. See Supplementary Materials Section S5 for more detail.

### 2.4. Sensitivity Analysis

#### 2.4.1. Global and Local Parameter Sensitivity

We used derivative-based and elementary effect frameworks to explore the sensitivity of model states to changes in parameter values. Specifically, Morris screening was chosen as the global sensitivity tool to explore the parameter space as a whole, while local sensitivity is conducted using a derivative-based method to focus on a specific fixed parameter set [30,31,32,33]. Morris screening determines a rank value for the sensitivity of each model state with respect to individual parameters by sampling from the entire parameter space. The ranking of Morris screening is a qualitative method of ordering parameters from the least to most influential in order to identify the subset of model parameters that have the least or no effect on the model output [30]. When a parameter has a low ranking or no effect on a desired state, this indicates that accurate estimation of the nominal parameter value from data is unlikely.

Unlike global sensitivity, the derivative-based local sensitivity method explores the sensitivity of the model states with respect to the parameters for only a single set of values [34,35]. This provides a quantitative measure to compare the influence of the parameters on a given model and, unlike a ranking system, their values can be used to draw conclusions about the magnitude differences between the sensitivity values [30]. Mathematical details of the analysis are found in Supplementary Materials S3. The viral load *V* is the primary variable of interest, because clinical BKPyV measurements are available, and the amount of BKPyV directly affects four other model states; however, sensitivity analysis is also conducted on the creatinine compartment, *C*. By determining which parameters affect these states, the sensitivity analysis assists parameter estimation, allowing us to fix some of the parameters to nominal values. If large changes in a parameter have only a small effect on the measured state variables, those parameters will have no influence on fitting the model to patient data.

#### 2.4.2. Sensitivity to Data Changes

Additionally, for CTOT patient #287915, we test the sensitivity of model 2 to changes in the data. We synthesize an observation of BKPyV viral load between the patient’s actual tests and re-fit the model with the synthesized data point included.

#### 2.4.3. Sensitivity to Drug Efficacy

Drug efficacy is the sole means of controlling the system. For parameter estimation, we hold ϵ constant. However, we also vary ϵ to demonstrate that changing drug dosage (and thus efficacy) affects the severity of BKPyV infection.

### 2.5. Identifiability

Identifiability refers to the ability to uniquely infer parameter values from data [33,34]. If parameters are not identifiable, more than one parameterization can generate the same observations from the model. To determine the identifiability of the parameters the model is sensitive to, eigenvalues of the sensitivity matrix are calculated. The sensitivity matrix consists of the partial derivatives found in the local sensitivity analysis; details of the algorithm are in Supplementary Materials S4.

### 2.6. Equilibria and Stability

With the assumptions that all parameters must be positive, we calculate equilibria by setting Equation (1) through (6) to 0 and solving [36]. Linear stability analysis determines the stability at each equilibrium point. See Supplementary Materials S5 for calculations of the equilibria.

## 3. Results

### 3.1. Model Development

This model is a simplification of the immune response model developed by one of the authors (Tran) [17]. We have increased the parsimony of the original model, reducing the number of parameters from 20 to 14, not including our immunusuppression term ϵ. Our results demonstrate that parsimony is not at the expense of goodness of fit. A full description of the model simplification is included in the Supplementary Materials (Section S2).

### 3.2. Parameter Estimation

Both models 1 and 2 are fit to EHR patient data, and we compare model results using the sum of the squared residuals (SS). The best fit trajectories of model states as functions of time for patients EHR #4080, CTOT #287915, EHR #4947, CTOT #287779, and CTOT #287660, are shown in Section 3.2 and Section 3.5, and Supplementary Materials S1. Viral load data are used to fit all trajectories; following this, creatinine data are then used to fit the creatinine trajectory. In the figures, data are given by black x’s, the model 1 state solutions are purple (when available), and those of model 2 are blue. Table 5 gives the SS for each set of patient data; the lower the SS, the better the model fit. While model 1 has lower residuals than model 2, the fits to data are comparable, and recall that model 2 is simpler and more biologically faithful.

**Figure 3:**
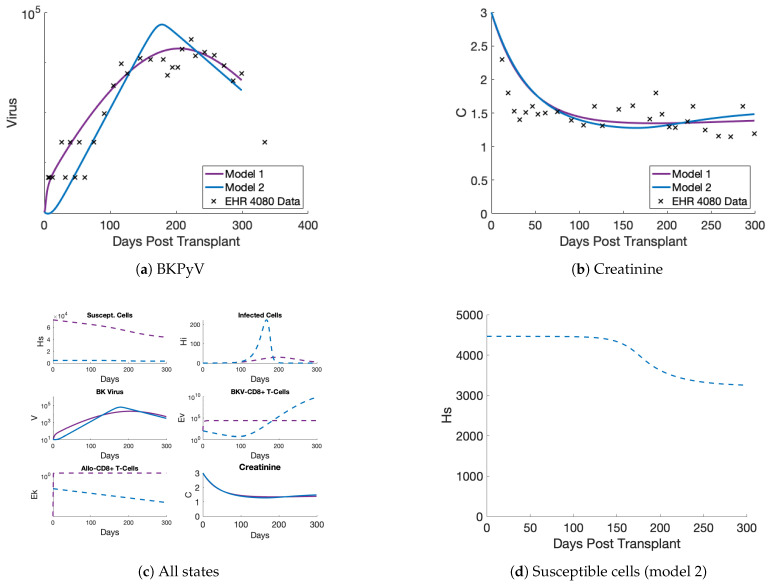
Model fits to EHR patient #4080 data. (**a**) Model 2 captures the BKPyV increase in the non-censored data better than model 1 and captures the decrease. (**b**) Both models fit the patient creatinine data equally well. (**c**) The unobserved model states (dashed lines) are shown here, which the model simulates without corresponding data. (**d**) The susceptible cell dynamics of model 2 show a plateau effect and stabilization of the allograft once a BKPyV infection is under control. This figure is a zoomed-in effect of the top left panel of (**c**).

Focusing on the model 2 fit in Figure 3, we see that the model fits BKPyV and creatinine data well. The model captures the climb in viral load around 100 days post-transplantation, when we have our first measurement over the limit of detection. (Note that there are two limits of detection, corresponding to different laboratories’ analyses of samples.) The model does over-estimate the peak viral load, but the tail of the infection is captured well. The creatinine state falls, as expected, immediately after transplant when the new kidney clears creatinine efficiently, and after a few months, the creatinine dynamics flatten out. Figure 3c shows all the states of the model after being fit to BKPyV and creatinine measurements. In the top left panel, notice that susceptible kidney cells ($H_{S}$) start at different amounts for the models due to the estimation of the initial value ($H_{S0}$). (This is acceptable, since susceptible cell concentrations of a donor organ are unknown and initial cell concentrations vary greatly among adults [37].) Behavior of the dynamics for $H_{S}$ are more important than the quantity of susceptible cells. In contrast to model 1’s (purple) steady fall of $H_{S}$ in Figure 3c, Figure 3d zooms in onmodel 2 (blue), showing a more stable susceptible cell population as it plateaus. Slow loss and a fairly level trajectory for susceptible cells indicates longer graft life. Pairing this behavior with the low constant creatinine level indicates the organ is still healthy using model 2. Also, the infected cells and BKPyV-specific T cells on the right-hand side follow the viral increase as expected. Overall, model 2 performs better than model 1 in the non-viral states while also being a simpler, more biologically focused model than previous versions.

**Figure 4:**
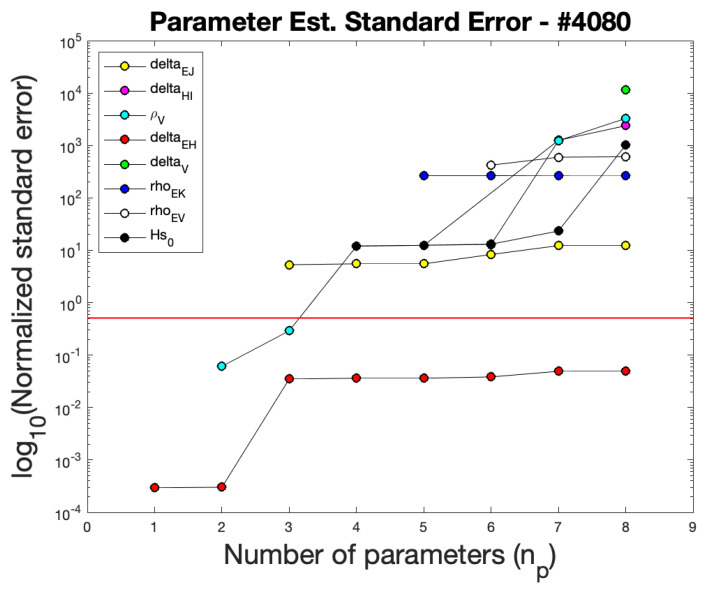
Parameter estimate subset selection based on standard errors for patient #4080. Each number of parameters indicates the best standard errors for a subgroup of that size. The red line indicates where standard errors are greater than their respective parameter values. For patient #4080, at best two parameters can be estimated with acceptable standard errors: $\delta_{EH}$ and $\rho_{V}$. Estimating all eight parameters, as shown in Table 6, only has a single parameter with respectable standard error.

In Table 6, the values for the parameters are listed that produced the model fit in Figure 3. The results of calculating normalized standard errors for the patient #4080 parameter estimates are displayed in Figure 4. Here, standard errors below the red threshold indicate that the standard errors are below the value of the parameter, and represent a relatively good estimate of the parameter value. If the number of parameters estimated were at two or three, Figure 4 shows that both $\delta_{EH}$ and $\rho_{V}$ would have reasonable standard errors. For the case of estimating all eight parameters together (as was performed for this work), the standard errors for all parameters except $\delta_{EH}$ exceed the threshold. Standard errors above the threshold are larger than the estimated value, and this result indicates a high likelihood that many of the parameters are correlated. This is not unrealistic for a nonlinear model such as the one presented in this work. Due to the likely correlation of the model parameters, determining parameter estimates with low standard error is not our focus. Overall, our goal is to produce acceptable model fits to patient data, and the model achieves this. Standard error figures for the other model fits are included in Supplemental Materials Section S5.

### 3.3. Sensitivity Analysis

**Figure 5:**
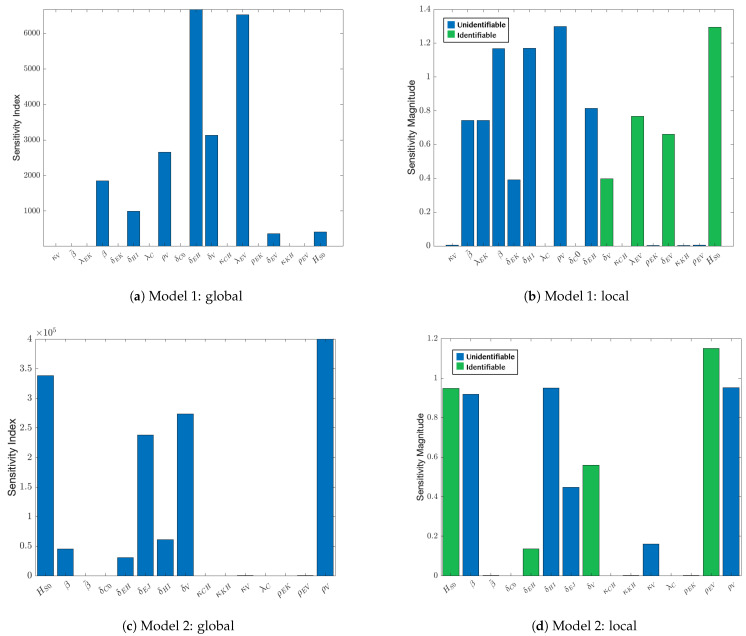
Sensitivity analysis for models 1 and 2. (**a**) The global analysis of model 1 shows half of the parameters have limited or no influence on BKPyV dynamics for a wide range of patients. (**b**) For patients in this study, the local analysis indicates the relative importance each parameter plays on BKPyV dynamics. There are four parameters that are uniquely identifiable from data in the model. (**c**) For model 2, the global sensitivity suggests that, again, for a wide range of patients, approximately half of the parameters have limited influence on BKPyV, similar to part (**a**). (**d**) The local sensitivity for model 2 shows that after improvements to the model, four parameters are still identifiable. Green sensitivities are identifiable parameters.

Sensitivity results for model 1 are shown in Figure 5a,b, and results for model 2 are shown in Figure 5c,d. For model 1, the local and global sensitivity analyses were in agreement with regard to several parameters. Notably, the model is insensitive to $\kappa_{V}$, $\lambda_{C}$, $\delta_{C0}$, $\kappa_{CH}$, $\rho_{EK}$, $\kappa_{KH}$, $\rho_{EV}$. Of the 18 parameters considered (including the initial condition for the state $H_{S}$), small changes to 11 of them indicate an influence on the BKPyV state (Figure 5b) and could be estimated from the patient data. Note that green sensitivities represent identifiable parameters as discussed in Section 3.4.

In model 2, there are nine parameters locally and seven parameters globally that the BKPyV model state is sensitive to. Comparing the sensitivity results between models, the number of influential parameters is reduced in model 2, and the growth rate of the virus-activated T cells, $\rho_{EV}$, has taken on the influential role from the model 1 T cell migration parameter $\lambda_{EV}$. Also, note that three of the parameters the model is not sensitive to (both locally and globally) are directly related to serum creatinine in Equation (6). This is expected as *C* directly depends on only the healthy kidney cells. Sensitivity analysis of *V* shows that these parameters cannot be estimated by BKPyV observations; however, using *C* as the state variable for sensitivity analysis shows that these parameters can be estimated using creatinine data.

Since both models have many parameters, the parameters that the virus compartment is not sensitive to are chosen to be fixed since changing them will have little to no effect on the model output. These parameters include $\kappa_{V}$, $\lambda_{C}$, $\delta_{C0}$, $\kappa_{CH}$, $\rho_{EK}$, and $\kappa_{KH}$, $\rho_{EV}$ for model 1, which are fixed to the values in Table 2 during BKPyV-related parameter estimation. For model 2, $\kappa_{V}$, $\lambda_{C}$, $\delta_{C0}$, $\kappa_{CH}$, and $\kappa_{KH}$ were chosen to be fixed, similar to model 1. Note that $\rho_{EK}$ is not included in this list. Since it is the only parameter to effect the growth of allo-specific effector cells, it is estimated. Initial estimates of $\tilde{\beta }$ and β discovered that the range where they produce biologically reasonable results was very narrow. Hence, both $\tilde{\beta }$ and β were fixed to the values in Table 2 for both models. We did not estimate $\kappa_{V}$, $\kappa_{KH}$, and $\kappa_{CH}$ because the model states were not sensitive enough to the half-saturation constant parameters.

The half-saturation terms are chosen to limit immune system growth as a specific threat decreases. These terms serve as throttles to the growth terms like $\rho_{EV}$. For example, in Equation (4), we see the term:

$$\frac{\rho_{EV}V}{V+\kappa_{V}}E_{V}$$

where the half-saturation expression $\frac{V}{V+\kappa_{V}}$ determines what proportion of the maximum growth rate $\rho_{EV}$ is active based on the magnitude of viral concentration. It is reasonable to assume that the patient’s immune response to the virus will be close to $\rho_{EV}$ when $V=10^{4}$ since BKPyV-induced nephropathy becomes a greater concern for a patient above $10^{4}$ [38]. By setting $\kappa_{V}=2500$, the *V*-dependent growth will be greater than or equal to 80% of $\rho_{EV}$ when $V\geq 10^{4}$. Similar reasoning was utilized in choosing $\kappa_{KH}$ in response to the amount of susceptible tubule epithelial cells.

### 3.4. Identifiability

Identifiable parameters are shown in green in the graphs on the right side of Figure 5. Note that our parameter identification algorithm relies on the local sensitivity analysis, so no green bars appear in Figure 5a,c. The set of identifiable parameters for model 1 are $\delta_{EV}$, $\delta_{V}$, $\lambda_{EV}$, $H_{S0}$, shown in green in Figure 5b. The identifiable parameters for model 2 are $H_{S0},\delta_{EH},\delta_{V},\rho_{EV}$. These are not the same parameters that are identifiable in model 1, but both model 1 and model 2 have four identifiable parameters, so there is no loss of identifying information induced by the model changes.

### 3.5. Sensitivity to New Data

**Figure 6:**
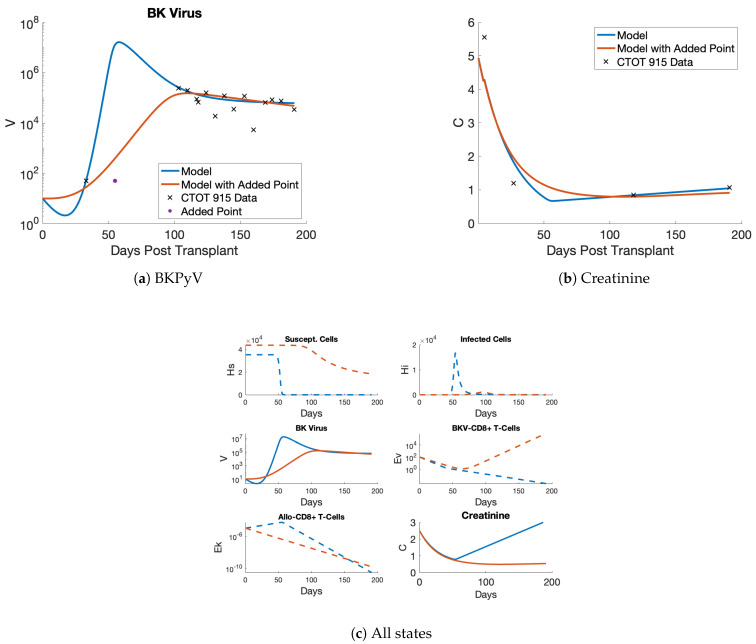
Model 2 fit to CTOT patient #287915 data. (**a**) The original model fit (blue) to the data displays a very early and strong BKPyV infection; the results of which would most likely lead to BKPyV nephropathy (BKPyVAN). For the model fit in red, a simulated data point is added to the dataset and results in more biologically realistic dynamics. (**b**) Limited creatinine data were available, but they are captured well by both models. (**c**) All states of the model are shown with both trajectories, where those in blue demonstrate an allograft lost to BKPyVAN, and others in red show a damaged but still functioning organ.

Patient #287915 has BKPyV data available to approximately 500 days post-transplant, but there is a large gap after day 200, so only the first approximately 200 days of data are considered. In Figure 6, observe that this patient has an earlier peak than most other patients shown in this work. Also, notice that there is a large gap between the only data point where the virus is undetectable around day 30 post-transplant and the first detectable data point around day 100.

When fitting the model to this patient’s data, we initially obtained the blue curves in Figure 6a. Note that this fit jumps up to a very high viral load quickly and decimates the allograft in the process. (See Figure 6c, top left panel, where susceptible cells hover close to zero for the blue fit.) This viral trajectory is unreasonable given that this patient remained in the clinical trial and did not receive a second transplant. A logical assumption follows that with more data between days 30 and 100, this trajectory would be different. To address this assumption, the red viral trajectory in Figure 6a is obtained by inserting a simulated data point at limit of detection on day 55. Other patient data observed in Figure 3 include data that lie at the limit of detection up to day 100. Our choice of simulated data point is supported by these data. This new fit (red trajectory) not only captures the remaining data but also maintains a healthy kidney as opposed to the blue model fit.

While we acknowledge that data collection and availability has greatly improved over the past decade, not all datasets have high fidelity. Mechanistic modeling is aided by rich datasets that do not have such gaps. We demonstrate here that modeling renal transplant patient dynamics accurately can be achieved with simulated data if the gap occurs prior to the patient’s first data point above the limit of detection.

### 3.6. Sensitivity to Drug Efficacy

**Figure 7:**
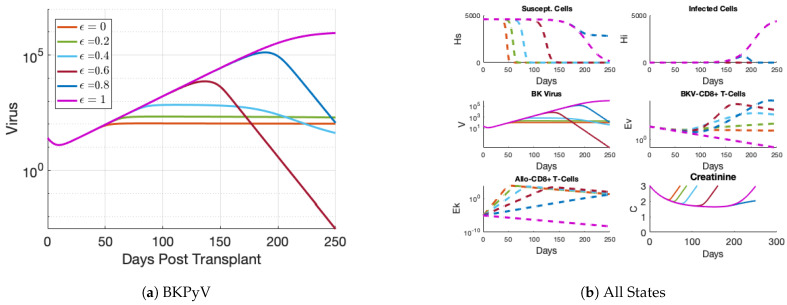
The effect of immunosuppression efficacy value (ϵ) on model fits to EHR patient #4947 data. (**a**) Reducing the efficacy of the immunosuppression treatment from 1 to 0 limits the strength of the BKPyV infection. (**b**) The effect of immunosuppression efficacy on the other states shows that the balanced immunosuppression approach of e=0.8 maintains a healthy graft. The healthy graft is visible through the non-zero $H_{S}$ trajectory (**top left** panel) and extended creatinine trajectory below 3 (**bottom right** panel).

With the purpose of developing and calibrating the model, we chose a simple fixed value for the drug efficacy parameter, ϵ. In Figure 7, the parameter was varied over six fixed values starting at 0 (no immune suppression) and ending at 1 (complete immune suppression). For the model to achieve the viral loads observed in patient data (greater than $10^{4}$), ϵ greater than 0.6 was necessary; see Figure 7a. Choosing ϵ=0.8 slows the loss of the susceptible tubule cell concentration shown in the top left panel of Figure 7b indicating a close-to-balanced treatment level. This also corresponds with clinicians’ approach of prescribing high-efficacy immunosuppression treatments following the transplantation to prevent acute rejection of the graft.

### 3.7. Equilibria and Stability

With positive parameters and fixed, positive efficacy, the model has two equilibria, which are developed in detail in Supplementary Materials Section S5. One equilibrium point contains negative state values, so it is not biologically possible. Another equilibrium exists with the assumption that the system contains no virus *and* no graft-activated T cells, $E_{K}=V=0$. This additional constraint leads to the equilibrium point $\left({\bar{H}}_{S},0,0,0,0,\bar{C}\right)$. Here, $\bar{C}$ depends only on the value of ${\bar{H}}_{S}$ when all other quantities are extremely low or not present:

$$\bar{C}=\frac{\lambda_{C}}{\delta_{C}0}\left(\frac{{\bar{H}}_{S}+\kappa_{CH}}{{\bar{H}}_{S}}\right).$$

Since ${\bar{H}}_{S}$ is positive, $\bar{C}$ is also positive. Note that this equilibrium can be observed in healthy individuals where there is no foreign kidney tissue in the body and their BKPyV is maintained in a latent state. However, this equilibrium is clinically improbable since there is neither BKPyV in the system, nor a host T cell response to the donor kidney.

Renal transplant patients experiencing a reactivation of BKPyV and a fixed immunosuppression treatment efficacy are highly unlikely to achieve this equilibrium with a healthy allograft according to model 2. Using a fixed treatment efficacy, the driving forces to reduce $E_{V}$ and $E_{K}$ to zero are *V* or $H_{S}$, respectively. The half saturation terms which are part of their growth in Equations (4) and (5) require *V* to be close to zero to reduce $E_{V}$ to zero and $H_{S}$ to approach zero to drive $E_{K}$ to zero. Ultimately, unless it is assumed that $E_{K}$ is close to zero, $H_{S}$ must be reduced so much that nephropathy occurs in order to bring allo-specific CD8+ T cells down to zero for the equilibrium. So it could be possible to achieve this equilibrium, but $\bar{H_{S}}$ would be so low that, biologically, the patient would need either dialysis or a new kidney transplant. A way to help drive the system to this equilibrium without the occurrence of nephropathy is to have dynamic treatment efficacy through the use of an optimal control, as proposed by Banks et al. in their previous work [16,17].

We use the parameters in Table 6 to conduct a local stability analysis of this equilibrium. The linear stability analysis method is performed and categorizes the equilibrium as a saddle point for each of the patients examined in this paper. Saddle points are unstable equilibria, but one where there exists a trajectory that does lead to the equilibrium $\left({\bar{H}}_{S},0,0,0,0,\bar{C}\right)$. This coincides with the description of possibly attaining the equilibrium above. For more detail on linear stability analysis and the model equilibria, see Supplementary Materials S5 and [39] for reference.

## 4. Discussion

Mechanistic modeling of immunosuppression in renal transplant patients is a crucial step in the development of a tool that provides optimal immunosuppression recommendations. This will allow clinicians to make treatment decisions that are personalized to each patient. Our improvements to the immune response model for BKPyV-infected renal transplant patients promise better performance in an optimal control application due to its simplicity. When constructing mechanistic mathematical models, it is best practice to follow the biology while employing the simplest model that captures the necessary dynamics. Each of the changes made to model 1 are based on biological criteria while also reducing the complexity in the model.

Local and global sensitivity analyses indicated which parameters are the least influential to the dynamics and guided the selection of parameters to estimate with patient data. While major changes were implemented to the Banks et al. immunosuppression model [16] in previous work [17,19], those works did not include a sensitivity analysis. A comparison of the analyses shown in Figure 5 provides information to separate our parameters into fixed, or “population level” parameters and those that must be determined for each individual patient.

With population-level parameters fixed, model fits to data through the estimation of individual specific parameters allows personalization of the model. Personalization is crucial, as patients can have very different responses to changes in immunosuppressant regimens.

The success of fitting the model to individuals as shown in Figure 3, Figure 6 and Figures S1–S3 indicates the model can be used to predict the viral and creatinine dynamics observed in the subset of real patients with peak BKPyV loads between 100 and 300 days post-transplant with viral peaks between $10^{4}$ and $10^{6}$ copies per mL. This is the first calibration of an immune response model for BKPyV-susceptible renal transplant patients since 2012 [40].

We have demonstrated that the model can be calibrated to a representative subset of renal transplant patients. However, we acknowledge that the $E_{K}$ state dynamics do not behave as well as we would like and are lower than expected. It is a challenge to obtain accurate immune response behavior both from the allo-specific and BKPyV-specific effector cells in the model since data for these responses are not collected as part of routine checkups. Flow cytrometry assays do exist for detecting the amount of specific effector cells present within a sample and making collection of these data routine for renal transplant patients would enhance parameter estimation for our model [41]. Collection of additional effector data may also allow us to incorporate other immune responses (e.g., neutralization or antibody-dependent cellular cytotoxicity) into the model, as necessary. It is also possible that our constant drug efficacy assumption is too restrictive. Future work using maintenance drug therapy data and other immune function indicators, such as CMV or EBV levels, to estimate a dynamic efficacy could resolve this issue. Additionally, future work will include personalizing immunosuppression treatment for the current subset of patients by applying the optimal control theory and a receding horizon control to Model 2, similar to the work in [17,19,20].

Personalized medicine is of growing interest in many areas of healthcare and we facilitate that by improving the foundation of the Banks et al. model [17]. Our work is novel given that mechanistic modeling is quite understudied in the subject of immune response in renal transplant patients [42]. Traditional clinical studies employ statistics to measure population effects, while our mathematical model captures individual disease progression. Also, the mechanistic modeling approach is a tiny part of the mathematical modeling of the allograft survival literature, where machine learning dominates the bulk of studies [43,44,45,46]. In contrast to machine learning methods, our approach to modeling the effects of BKPyV and immune response in allograft survival provides the opportunity for further understanding of the symbiosis between the allograft, the host immune system, and renal-focused pathogens. This is a key benefit of mechanistic modeling since the model directly describes interactions between the parts of the biological system and the rates of change (parameters) which guide them, as opposed to “black box” machine learning models. As shown in Figure 3, Figure 6 and Figures S1–S3, we can observe the predicted dynamics in each of the biological components and recognize how changes to the parameters during estimation influence those dynamics. This provides the insight necessary to increase model complexity where it is necessary to accurately model the biological system of a renal transplant patient undergoing immunosuppression treatment. By providing researchers and clinicians the ability to see the predicted interactions between the biological components in the model as it advances in complexity, they may observe unexpected behavior in the model dynamics, which can lead to new experiments and advancing the understanding of balancing immunosuppression in renal transplant patients. Mechanistic modeling is an important part in the development of personalized medicine and optimizing immunosuppression treatment for renal transplant patients.

## 5. Conclusions

The work presented here improves upon an existing immune response model for BKPyV-susceptible renal transplant recipients by reducing the complexity of the model while maintaining the model’s ability to capture the dynamics of the BKPyV and serum creatinine levels found in the data. The new model was successfully calibrated with real clinical data of five renal transplant recipients taken from the Duke Transplant Center EHR and NIH CTOT datasets. Including the sensitivity analyses of the model provides insight into the types and qualities of the parameters in the model, which focuses the scope of the parameter estimation for a more efficient model calibration process.

This demonstrated success in the initial calibration of the model using real patient data paves the way forward for advances in modeling data-supported optimal control of immunosuppression. An important next step should add dynamic immunosuppression into the model by adding common drug treatment dynamics and calibrating the model with drug data from additional patients in the full datasets. This work supports our ultimate goal and brings us one step closer to providing a tool for clinicians which predicts the outcomes of different treatment strategies and assists in prescribing personalized optimal immunosuppression treatments for individual patients.

## Figures and Tables

**Table 1:** State variables in the mathematical model.

State	Description	Unit
$H_{S}$	Density of susceptible graft cells	cells/mL
$H_{I}$	Density of infected graft cells	cells/mL
*V*	Concentration of free BKPyV	copies/mL
$E_{V}$	Concentration of BKPyV-specific CD8+ T-cells	cells/mL
$E_{K}$	Concentration of allo-specific CD8+ T-cells	cells/mL
*C*	Concentration of serum creatinine	mg/dL

**Table 2:** Initial values of parameters for model 2.

Parameter	Value	Description	Units
β	$8.22\times 10^{-8}$	Infection rate of $H_{S}$ by V	mL/(copies·day)
$\tilde{\beta }$	0.0001	Attack rate on $H_{S}$ by $E_{K}$	mL/(cells·day)
$\delta_{HI}$	0.085	Death rate of $H_{I}$ by V	/day
$\delta_{EH}$	0.0018	Elimination rate of $H_{I}$ by $E_{V}$	mL/(cells·day)
$\rho_{V}$	15,000	Virions produced by $H_{I}$ before death	copies/cells
$\delta_{V}$	0.05	Natural clearance rate of V	/day
$\rho_{EV}$	0.36	Maximum proliferation rate for $E_{V}$	/day
$\kappa_{V}$	2500	Half saturation constant	copies/mL
$\delta_{EJ}$	0.17	Death rate of $E_{V}$ and $E_{K}$	/day
$\rho_{EK}$	0.137	Maximum proliferation rate for $E_{K}$	/day
$\kappa_{KH}$	$10^{3}$	Half saturation constant	cells/mL
$\lambda_{C}$	0.01	Production rate for C	mg/(dL·day)
$\delta_{C0}$	0.2	Maximize clearance rate for C	/day
$\kappa_{CH}$	$10^{4}$	Half saturation constant	cells/mL

Default values for Model 2 parameters. These values come from a previous model version in [17], $\kappa_{V}$ comes from Section 3.3, and $\delta_{EJ}$ from the development of model 2 in Supplementary Materials Section S2.

**Table 3:** Duke EHR data summary.

Characteristic	n = 443
Unusable data (sparse/below LOD)	191 (43.1%)
Peak above LOD but below $10^{4}$	132 (29.8%)
**Peak BKPyV above $10^{4}$ and peaks before day 300**	**92 (20.7%)**
Peak BKPyV above $10^{4}$ and peaks after day 300	28 (6.3%)

Limit of Detection (LOD) is 50 copies/mL. Sparse data consist of any patient with less than 10 viral measurements. For this work, we consider the patients with peak viral loads above $10^{4}$ before day 300.

**Table 4:** CTOT-19 data summary.

Characteristic	n = 220
Unusable data (sparse/below LOD)	172 (78.2%)
Peak above LOD but below $10^{4}$	19 (8.6%)
**Peak BKPyV above $10^{4}$ and peaks before day 300**	**22 (10%)**
Peak BKPyV above $10^{4}$ and peaks after day 300	7 (3.2%)

Limit of Detection (LOD) is 50 copies/mL. Sparse data consist of any patient with less than 10 viral measurements. For this work, we consider the patients with peak viral loads above $10^{4}$ before day 300.

**Table 5:** BKPyV residuals for the model fits using log scale.

Patient	Model	BKPyV Residual
EHR 4080	1	3.5565
	2	7.2772
EHR 4947	1	2.7369
	2	3.1951
CTOT 779	2	1.5626
CTOT 915	2	2.1032
CTOT 660	2	5.9411

**Table 6:** Parameter estimates for each patient.

Parameter	EHR 4080	EHR 4947	CTOT 779	CTOT 915	CTOT 660
β	$8.22\times 10^{-8}$	$8.22\times 10^{-8}$	$8.22\times 10^{-8}$	$8.22\times 10^{-8}$	$8.22\times 10^{-8}$
$\tilde{\beta }$	0.0001	0.0001	0.0001	0.0001	0.0001
$\delta_{HI}$	$3.190\times 10^{-5}$	$1.156\times 10^{-4}$	$3.121\times 10^{-5}$	$6.845\times 10^{-4}$	$2.818\times 10^{-4}$
$\delta_{EH}$	$2.661\times 10^{-5}$	$3.581\times 10^{-6}$	0.069	0.294	$1.409\times 10^{-3}$
$\rho_{V}$	$4.075\times 10^{5}$	$2.625\times 10^{5}$	$4.94\times 10^{5}$	$4.242\times 10^{5}$	$6.548\times 10^{5}$
$\delta_{V}$	0.0260	0.1430	0.0248	0.1697	0.1854
$\rho_{EV}$	0.939	0.692	0.877	0.344	0.275
$\kappa_{V}$	2500	2500	2500	2500	2500
$\delta_{EJ}$	0.0422	0.0300	0.0748	0.1064	0.0306
$\rho_{EK}$	0.00612	0.5477	0.3241	0.7064	0.4582
$\kappa_{KH}$	$10^{3}$	$10^{3}$	$10^{3}$	$10^{3}$	$10^{3}$
$\lambda_{C}$	0.0283	0.0381	0.3327	0.00284	0.1416
$\delta_{C0}$	0.07497	0.07674	0.2309	0.0507	0.9999
$\kappa_{CH}$	$10^{4}$	$10^{4}$	$10^{4}$	$10^{4}$	$10^{4}$
$H_{S}\left(0\right)$	4464	4577	20,978	35,317	5064

Parameters highlighted in grey are not estimated and parameters highlighted in red are fit to creatinine data separately. Other parameters and initial value for $H_{S}$ are fit to BKPyV data for each patient.

## Data Availability

The PROTOCOL CTOT-19 dataset is provided by the Clinical Trials in Organ Transplantation consortium sponsored by the National Institute of Allergy and Infectious Diseases via Immport. The patient data from the Duke Transplant Center electronic health record and code to generate the figures are available upon request.

## Supplementary Materials

The following supporting information can be downloaded at: https://www.mdpi.com/article/10.3390/v17010050/s1 [47,48,49,50,51,52,53,54,55,56,57,58,59], Section S1 contains additional Figures S1–S3, the model fits referred to in Section 3 for EHR patient #4947 and CTOT patients #287779 and #287660 respectively. Section S2 describes the iterative changes made to the model from versions 0 through 2. Model 2 is the Updated Immunosuppression Model described in Section 2. Table S1 contains the initial parameter estimates for model 1. Figure S4 displays model 1 fit to EHR #4947 data. Figure S5 demonstrates that changes to CD8+ T cell’s loss terms are beneficial to parameter estimation. Figure S6 is the model 2 diagram for reference for all model changes in Section S2. Section S3 details methodology for sensitivity analysis that produces Figure 5. Section S4 covers the mathematical process for parameter identifiability from Section 3.4 and also shown in Figure 5b,d. Section S5 discusses the standard errors for the parameter estimation similar to the end of Section 3.2 and provides standard error Figures S7 and S8 for the remaining patients. Section S6 calculates the model equilibria and describes long term dynamics with Tables S2, S3, S4, S5 and S6 showing the equilibria and eigenvalues from CTOT patients #287660, #287915, #287779 and EHR patients #4080 and #4947 respecitvely.

## References

[1] Kalluri H., Hardinger K. (2012). Current state of renal transplant immunosuppression: Present and future. World J. Transplant..

[2] Kant S., Dasgupta A., Bagnasco S., Brennan D.C. (2022). BK Virus Nephropathy in Kidney Transplantation: A State-of-the-Art Review. Viruses.

[3] Sellares J., De Freitas D., Mengel M., Reeve J., Einecke G., Sis B., Hidalgo L., Famulski K., Matas A., Halloran P. (2012). Understanding the causes of kidney transplant failure: The dominant role of antibody-mediated rejection and nonadherence. Am. J. Transplant..

[4] Funk G., Gosert R., Comoli P., Ginervri F., Hirsch H. (2008). Polyomavirus BK replication dynamics in vivo and in silico to predict cytopathology and viral clearance in kidney trans-plants. Am. J. Transplant..

[5] Perelson A.S., Neumann A.U., Markowitz M., Leonard J.M., Ho D.D. (1996). HIV-1 Dynamics In Vivo: Virion Clearance Rate, Infected Cell Life-Span, and Viral Generation Time. Science.

[6] Boianelli A., Nguyen V.K., Ebensen T., Schulze K., Wilk E., Sharma N., Stegemann-Koniszewski S., Bruder D., Toapanta F.R., Guzmán C.A. (2015). Modeling Influenza Virus Infection: A Roadmap for Influenza Research. Viruses.

[7] Rong L., Perelson A.S. (2010). Treatment of hepatitis C virus infection with interferon and small molecule direct antivirals: Viral kinetics and modeling. Crit. Rev. Immunol..

[8] Perelson A.S., Guedj J. (2015). Modelling hepatitis C therapy—Predicting effects of treatment. Nat. Rev. Gastroenterol. Hepatol..

[9] Perelson A.S., Nelson P.W. (1999). Mathematical Analysis of HIV-1 Dynamics In Vivo. SIAM Rev..

[10] West R., Crosby D., Jones J. (1974). A mathematical model of an integrated haemodialysis and renal transplantation programme. Br. J..

[11] Labert P., Collett D., Kimber A., Johnson R. (2004). Parametric accelerated failure time models with random effects and an application to kidney transplant survival. Stat. Med..

[12] Topuz K., Zengul F., Dag A., Almehmi A., Yildirim M. (2018). Predicting graft survival among kidney transplant recipients: A Bayesian decision support model. Decis. Support Syst..

[13] Bang J., Bae J., Oh C.K. (2020). Mathematical model for early functional recovery pattern of kidney transplant recipients using serum creatinine. Korean J. Transplant..

[14] Blazquez-Navarro A., Schachtner T., Stervbo U., Sefrin A., Stein M., Westhoff T.H., Reinke P., Klipp E., Babel N., Neumann A.U. (2018). Differential T cell response against BK virus regulatory and structural antigens: A viral dynamics modelling approach. PLoS Comput. Biol..

[15] Mahato H., Ahlstrom C., Jansson-Löfmark R., Johansson U., Helminger G., Hallow K. (2018). Mathematical model of hemodynamic mechanisms and consequences of glomerular hyper-tension in diabetic mice. NPJ Syst. Biol. Appl..

[16] Banks H.T., Hu S., Link K., Rosenberg E.S., Mitsuma S., Rosario L. (2016). Modelling immune response to BK virus infection and donor kidney in renal transplant recipients. Inverse Probl. Sci. Eng..

[17] Murad N., Tran H., Banks H. (2019). Optimal control of immunosuppressants in renal transplant recipients susceptible to BKV infection. Optim. Control. Appl. Methods.

[18] Murad N., Tran H.T., Banks H., Everett R.A., Rosenberg E. (2018). Immunosuppressant treatment dynamics in renal transplant recipients: An iterative modeling approach. Discret. Contin. Dyn. Syst. B.

[19] Murad N. (2018). Quantitative Modeling and Optimal Control of Immunosuppressant Treatment Dynamics in Renal Transplant Recipients. Ph.D. Thesis.

[20] Myers N.J. (2021). Applications of Mathematical Modeling in Ecology and Health Care. Ph.D. Thesis.

[21] Newell K. (2009). SDY1433: Optimization of NULOJIX^®^ (Belatacept) Usage as a Means of Minimizing CNI Exposure in Simultaneous Pancreas and Kidney Transplantation (CTOT-15).

[22] Heeger P. (2023). SDY2400: Effects of Inhibiting Early Inflammation in Kidney Transplant Patients (CTOT-19).

[23] Hirsch H., Randhawa P. (2019). BK polyomavirus in solid organ transplantation: Guidelines from the American Society of Transplantation Infectious Diseases Community of Practice. Clin. Transplant..

[24] Haldeman-Englert C., Cunningham Turley R., Novick T. Creatinine (Blood)—University of Rochester Medical Center. https://www.urmc.rochester.edu/encyclopedia/content.aspx?ContentTypeID=167&ContentID=creatinine_serum.

[25] Levey A.S., Stevens L.A., Schmid C.H., Zhang Y., Castro III A.F., Feldman H.I., Kusek J.W., Eggers P., Van Lente F., Greene T. (2009). A new equation to estimate glomerular filtration rate. Ann. Intern. Med..

[26] Bhattacharya S., Dunn P., Thomas C.G., Smith B., Schaefer H., Chen J., Hu Z., Zalocusky K.A., Shankar R.D., Shen-Orr S.S. (2018). ImmPort, toward repurposing of open access immunological assay data for translational and clinical research. Sci. data.

[27] Banks H., Bekele-Maxwell K., Canner J.E., Mayhall A., Menda J., Noorman M. (2017). The effect of statistical error model formulation on the fit and selection of mathematical models of tumor growth for small sample sizes. Int. J. Pure Appl. Math..

[28] The MathWorks Inc. (2022). MATLAB Version: 9.13.0 (R2022b).

[29] Banks H.T., Beraldi R., Cross K., Flores K., McChesney C., Poag L., Thorpe E. (2015). Uncertainty quantification in modeling HIV viral mechanics. MBE.

[30] Smith R.C. (2014). Uncertainty Quantification: Theory, Implementation, and Applications.

[31] Arthur J.G., Tran H.T., Aston P. (2017). Feasibility of parameter estimation in hepatitis C viral dynamics models. J. Inverse Ill-Posed Probl..

[32] Campolongo F., Saltelli A., Cariboni J. (2011). From screening to quantitative sensitivity analysis. A unified approach. Comput. Phys. Commun..

[33] Brady R., Frank-Ito D., Tran H., Janum S., Møller K., Brix S., Ottensen J., Mehlsen J., Olufsen M. (2018). Personalized mathematical model of endotoxin-induced inflammatory responses in young men and associated changes in heart rate variability. Math. Model. Nat. Phenom..

[34] Wentworth M.T., Smith R.C., Banks H. (2016). Parameter Selection and Verification Techniques Based on Global Sensitivity Analysis Illustrated for an HIV Model. SIAM/ASA J. Uncertain. Quantif..

[35] Saltelli A., Ratto M., Andres T., Campolongo F., Cariboni J., Gatelli D., Saisana M., Tarantola S. (2007). Introduction to Sensitivity Analysis. Global Sensitivity Analysis. The Primer.

[36] Otto S., Day T. (2007). A Biologist’s Guide to Mathematical Modeling in Ecology and Evolution.

[37] Bertram J.F., Douglas-Denton R.N., Diouf B., Hughson M.D., Hoy W.E. (2011). Human nephron number: Implications for health and disease. Pediatr. Nephrol..

[38] Borriello M., Ingrosso D., Perna A., Lombardi A., Maggi P., Altucci L., Caraglia M. (2019). BK Virus Infection and BK-Virus-Associated Nephropathy in Renal Transplant Recipients. Genes.

[39] Farlow J., Hall J.E., McDill J.M., West B.H. (2007). Differential Equations and Linear Algebra.

[40] Banks H.T., Hu S., Jang T., Kwon H.D. (2012). Modelling and optimal control of immune response of renal transplant recipients. J. Biol. Dyn..

[41] Enoksson S.L., Bergman P., Klingstrom J., Bostrom F., Rodrgues R.D.S., Winerdal M., Marits P. (2021). A flow cytometry-based proliferation assay for clinical evaluation of T-cell memory against SARS-CoV-2. J. Immunol. Methods.

[42] Fribourg M. (2020). A case for the reuse and adaptation of mechanistic computational models to study transplant immunology. Am. J. Transplant..

[43] Senanayake S., White N., Graves N., Healy H., Baboolal K., Kularatna S. (2019). Machine learning in predicting graft failure following kidney transplantation: A systematic review of published predictive models. Int. J. Med. Inform..

[44] Shaikhina T., Lowe D., Daga S., Briggs D., Higgins R., Khovanova N. (2019). Decision tree and random forest models for outcome prediction in antibody incompatible kidney transplantation. Biomed. Signal Process. Control.

[45] Udomkarnjananun S., Townamchai N., Kerr S.J., Tasanarong A., Noppakun K., Lumpaopong A., Prommool S., Supaporn T., Avihingsanon Y., Praditpornsilpa K. (2020). The first Asian kidney transplantation prediction models for long-term patient and allograft survival. Transplantation.

[46] Scheffner I., Gietzelt M., Abeling T., Marschollek M., Gwinner W. (2020). Patient survival after kidney transplantation: Important role of graft-sustaining factors as determined by predictive modeling using random survival forest analysis. Transplantation.

[47] Alcendor D.J. (2019). BK polyomavirus virus glomerular tropism: Implications for virus reactivation from latency and amplification during immunosuppression. J. Clin. Med..

[48] Janeway C.A., Travers P., Walport M., Shlomchik M.J. (2001). Immunobiology: The Immune System in Health and Disease.

[49] Antia R., Ganusov V.V., Ahmed R. (2005). The role of models in understanding CD8 + T-cell memory. Nat. Rev. Immunol..

[50] Ribeiro R.M., Mohri H., Ho D.D., Perelson A.S. (2002). In vivo Dynamics of T Cell Activation, Proliferation, and Death in HIV-1 Infection: Why Are CD4+but Not CD8+T Cells Depleted?. Proc. Natl. Acad. Sci. USA.

[51] Terry E., Marvel J., Arpin C., Gandrillon O., Crauste F. (2012). Mathematical model of the primary CD8 T cell immune response: Stability analysis of a nonlinear age-structured system. J. Math. Biol..

[52] Appay V., Rowland-Jones S.L. (2004). Lessons from the study of T-cell differentiation in persistent human virus infection. Semin. Immunol..

[53] Thomas-Vaslin V., Altes H.K., de Boer R.J., Klatzmann D. (2008). Comprehensive Assessment and Mathematical Modeling of T Cell Population Dynamics and Homeostasis1. J. Immunol..

[54] Campolongo F., Cariboni J., Saltelli A. (2007). An effective screening design for sensitivity analysis of large models. Environ. Model. Softw..

[55] Dennis J.E., Schnabel R.B. (1996). Numerical Methods for Unconstrained Optimization and Nonlinear Equations.

[56] Quaiser T., Mönnigmann M. (2009). Systematic identifiability testing for unambiguous mechanistic modeling–application to JAK-STAT, MAP kinase, and NF-*κ* B signaling pathway models. BMC Syst. Biol..

[57] Cintron-Arias A., Banks H., Capaldi A., Lloyd A. (2009). A sensitivity matrix based methodology for inverse problem formulation. J. Inverse Ill-Posed Probl..

[58] Banks H.T., Cintron-Arias A., Kappel F., Batzel J.J., Bachar M., Kappel F. (2013). Parameter selection methods in inverse problem formulation. Mathematical Modeling and Validation in Physiology.

[59] Bohl D.L., Brennan D.C. (2021). Matrix Methods: Applied Linear Algebra and Sabermetrics.

