# Supplementary material for: Modeling BK Virus Infection in Renal Transplant Recipients
Source: Viruses. 2024 Dec 31;17(1):50. doi: 10.3390/v17010050 (PMC11768487; doi:10.3390/v17010050)
Supplement: Supplementary file 1 [file viruses-17-00050-s001.zip › viruses-3316319-supplementary.pdf]

Supplemental Materials

S1. Additional Figures

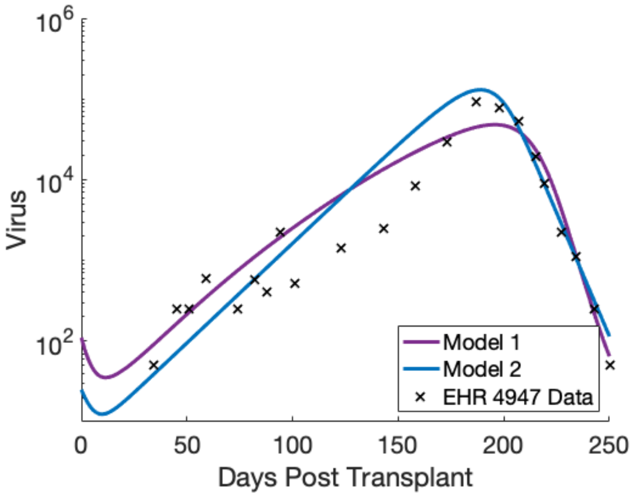

(a) BKPyV

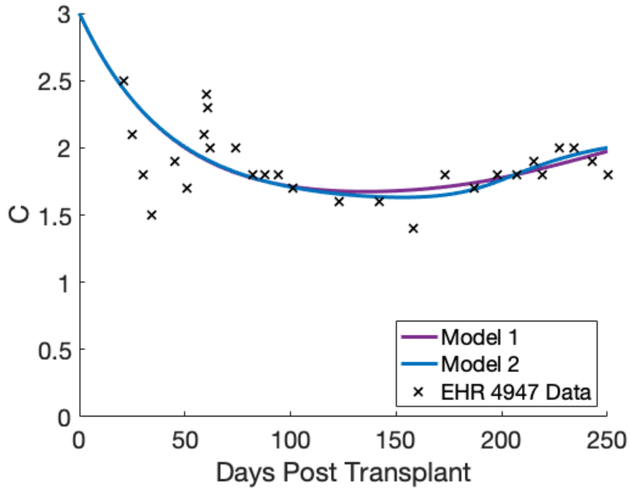

(b) Creatinine

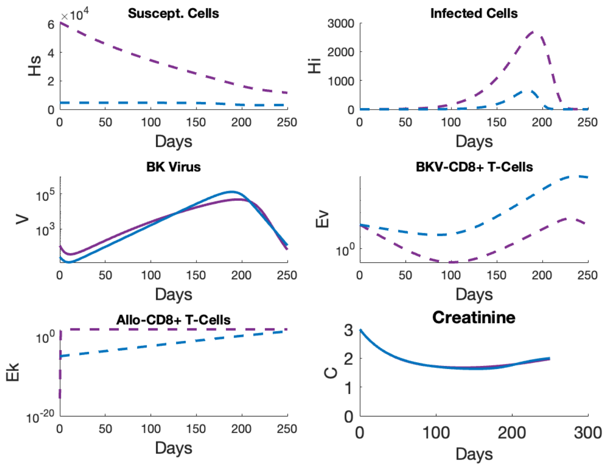

(c) All states

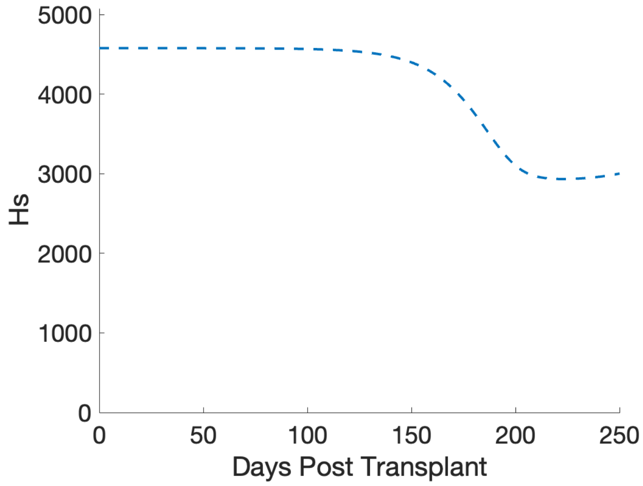

(d) Susceptible cells (model 2)

Figure S1. Model fits to EHR patient #4947 data. a) Model 2 fits to the peak of BKPyV data better than model 1. b) Model 1 and 2 fit the creatinine data equally well. c) The unobservable states help determine the effect of BKPyV in fection. The top left panel indicates the susceptible cells are in steady decline for model 1 due to the infection. With an approximate loss of 66% of these cells for model 1, there is likely graft impairment. d) A zoomed in plot of susceptible cells for model 2 due to the lower estimated initial value of H_S. The susceptible cells plateau indicating the stabilization of the graft once the infection is under control.

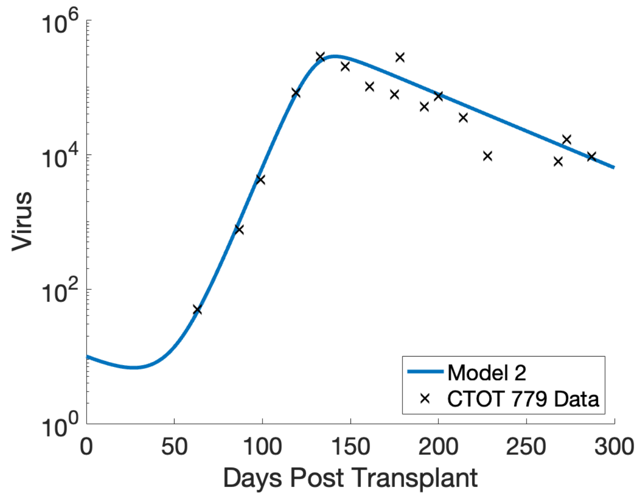

(a) BKPyV

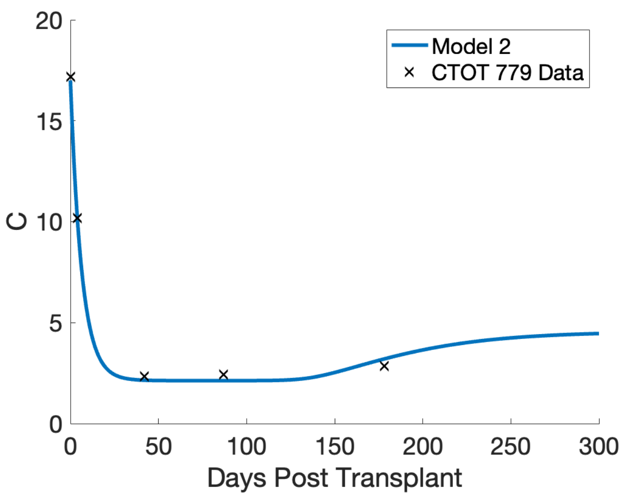

(b) Creatinine

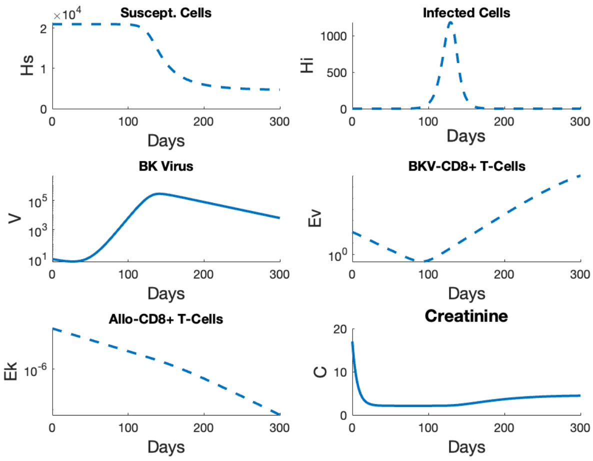

(c) All states

Figure S2. Model 2 fit to CTOT patient #287779 BKPyV data. a) Model 2 captures both the growth and decline of the patient's BKPyV data. The slow decline of BKPyV indicates the infection is not under control. b)Creatinine data is sparse but captured well c) Most important is the state H_S (top left panel) with a noticeable decline likely due to the persistent infection.

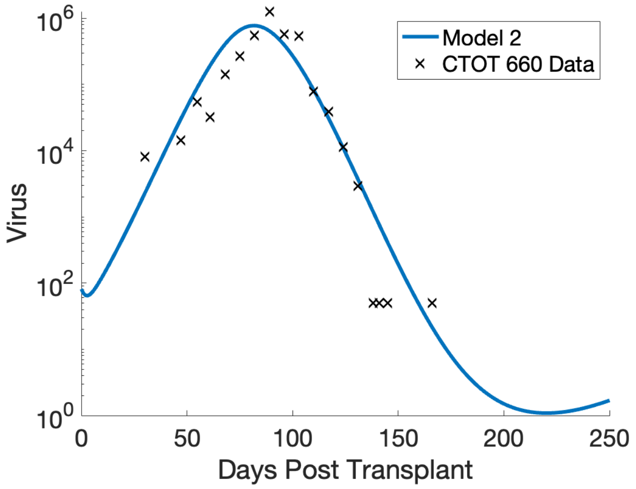

(a) BKPyV

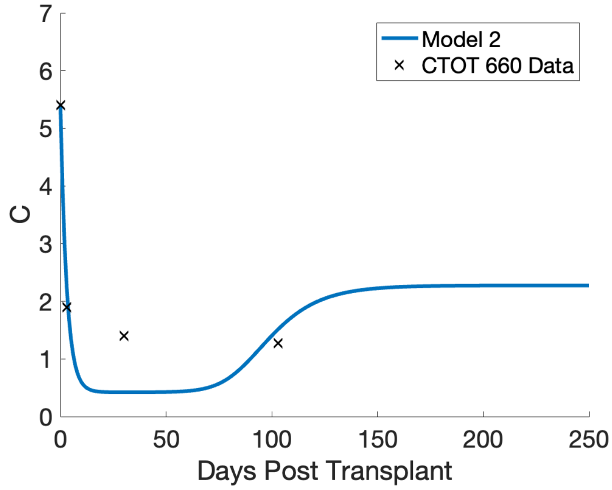

(b) Creatinine

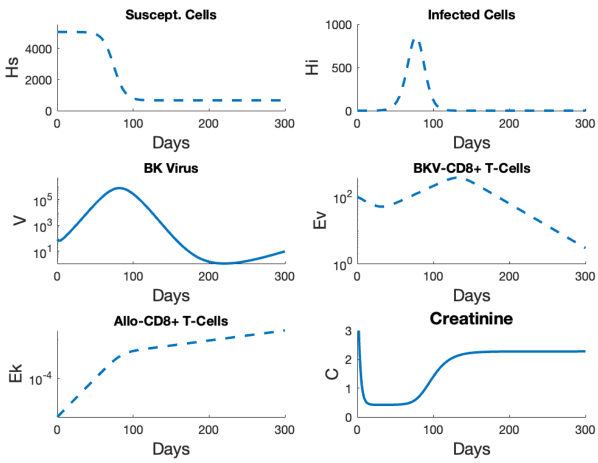

(c) All states

Figure S3. Model 2 fit to CTOT patient #287660 data. a) The model 2 fit to BKPyV data grows slightly quicker than the data and misses the high peak (10^6 copies/mL) but captures the decline of the virus very well. b) Creatinine data is too sparse to show if creatinine rises due to the loss of kidney around day 100 post transplant c) Susceptible cells (top left panel) decline significantly due to the excessive peak of BKPyV. The decline in H_S by 80% indicates almost total loss of kidney function which is supported by creatinine (bottom right panel) growing to 2 mg/dL.

S2. Model Improvements

We obtained our refined model using the sensitivity analyses and the method of model fitting described in the paper. The original model (model 0) was initially verified qualitatively by gathering and calculating parameters from literature, see [1] and [2]. There are 21 parameters in the mathematical model which include source and loss rates (δ_{EV}, ρ_V, etc.), half saturation constants (κ_{EH}, κ_V, κ_{CH}) and an immunosuppression efficacy value (ϵ). The model includes a set of characteristic functions, $\chi_{(V>V^*)}$, $\chi_{(E_K>E_K^*)}$, and $\chi_{(E_V>E_V^*)}$, with threshold parameters V^*, E_K^*, and E_V^* (see model 0 in Equations (1) - (6)). Certain elements of the model stood out as contrary to the biology or potentially unnecessary. We have examined this model and removed several parameters in the interest of making a simpler, more accurate model.

Immune Response Model: Model 0 [3]

$$\dot{H_S} = -\chi_{(V>V^*)}\beta H_S V - \chi_{(E_K>E_K^*)}\tilde{\beta} H_S E_K \quad (1)$$

$$\dot{H_I} = \chi_{(V>V^*)}\beta H_S V - \delta_{HI} H_I - \chi(E_V > E_{V^*})\delta_{EH} E_V H_I \quad (2)$$

$$\dot{V} = \rho_V \delta_{HI} H_I - \delta_V V - \chi_{(V>V^*)}\beta H_S V \quad (3)$$

$$\dot{E_V} = (1-\epsilon)\left(\lambda_{EV} + \frac{\rho_{EV} V}{V+\kappa_V} E_V\right) - \delta_{EV} E_V \quad (4)$$

$$\dot{E_K} = (1-\epsilon)\left(\lambda_{EK} + \frac{\rho_{EK} H_S}{H_S+\kappa_{KH}} E_K\right) - \delta_{EK} E_K \quad (5)$$

$$\dot{C} = \lambda_C - \delta_{C0}\frac{H_S}{H_S+\kappa_{CH}} C \quad (6)$$

Initially, characteristic functions are removed. The V^*, E_V^*, and E_K^* characteristic functions set the minimum quantities of BKPyV copies and CD8+ T cells in the blood before they could affect the body. In the case of BK virus, this was implemented as a representation of BKPyV in the latent state before reactivating when the threshold for viral copies in the blood (V^*) was met [2]. Instead, reactivated virions can be addressed in the estimated growth term for BKPyV so modeling BKPyV latency is unneeded. In the comprehensive account of BKPyV by Alcendor, et. al., we note there is no notion of a threshold to determine activation of BKPyV [4]. Similarly, the other characteristic functions, for E_K^* and E_V^*, indicate the effector cell populations will not attack their targets (susceptible cells and infected cells, respectively) until high enough concentrations were present in the blood. This is also not suggested in the literature [5]. By removing these expressions, CD8+ T cells are effective at controlling their targets immediately which would occur in the biological system when the cells come in contact. Previously, low viral data paired with the viral threshold parameter led to biologically unrealistic behavior in the dynamics and difficulty fitting parameters using OLS on account of the discontinuity from crossing the threshold. By removing the characteristic functions from the model, we remove this complication and are able to work with the low viral load and low effector cell levels common in many patients.This change also improved the model's performance numerically and reduced the size of the parameter space.

After removing the characteristic functions, we are left with model 1 in Equations (7)-(12) which could be compared to other refinements of the model. The estimated parameter values for model 1 are listed in Table S1. The model dynamics due to this change are shown in model 1 and for patient #4947 in Figure S4. Figure S4 is the result of the OLS fitting process outlined in section 2.3. (Note this change to the model was made before the decision to adjust the fixed values of κ_V and κ_{KH} as described in section 3.3.)

Even though this change allowed for better model fitting of viral data, some issues still exist in other states. Most important is that the susceptible renal tubule cell population declines severely with significant loss of kidney function being likely; see top left panel of Figure S4. In addition, bottom left panel shows the almost instantaneous growth of allo-specific effector cells to a carrying capacity of 50 cells and seems biologically unrealistic.

Table S1. Parameter estimates for model 1

Parameter	Value	Description	Units
β	$8.22x10^{-8}$	Infection rate of H_S by V	mL/(copies· day)
$\tilde{\beta}$	0.0001	Attack rate on H_S by E_K	mL/(cells· day)
δ_{HI}	0.085	Death rate of H_I by V	/day
δ_{EH}	0.0018	Elimination rate of H_I by E_V	mL/(cells· day)
ρ_V	15000	Virions produced by H_I before death	copies/cells
δ_V	0.05	Natural clearance rate of V	/day
λ_{EV}	285	Source rate of E_V	cells/(mL· day)
ρ_{EV}	0.36	Maximum proliferation rate for E_V	/day
κ_V	$\mathbf{10^7}$	Half saturation constant	copies/mL
δ_{EV}	0.17	Death rate of E_V	/day
λ_{EK}	285	Source rate of E_K	cells/(mL· day)
ρ_{EK}	0.137	Maximum proliferation rate for E_K	/day
κ_{KH}	$\mathbf{10^5}$	Half saturation constant	cells / mL
δ_{EK}	0.09	Death rate of E_K	/day
λ_C	0.01	Production rate for C	mg/(dL· day)
δ_{C0}	0.2	Maximize clearance rate for C	/day
κ_{CH}	10^4	Half saturation constant	cells/mL

Parameters highlighted in grey are not estimated, parameters highlighted in red are fit to creatinine data separately, and other parameters and initial value for HS are fit to BKPyV data.

Immune Response Model: Model 1

$$\dot{H_S} = -\beta H_S V - \tilde{\beta} H_S E_K \tag{7}$$

$$\dot{H_I} = \beta H_S V - \delta_{HI} H_I - \delta_{EH} E_V H_I \tag{8}$$

$$\dot{V} = \rho_V \delta_{HI} H_I - \delta_V V - \beta H_S V \tag{9}$$

$$\dot{E_V} = (1-\epsilon)\left(\lambda_{E_V} + \frac{\rho_{EV} V}{V + \kappa_V} E_V\right) - \delta_{E_V} E_V \tag{10}$$

$$\dot{E_K} = (1-\epsilon)\left(\lambda_{E_K} + \frac{\rho_{EK} H_S}{H_S + \kappa_{KH}} E_K\right) - \delta_{E_K} E_K \tag{11}$$

$$\dot{C} = \lambda_C - \delta_{C0} \frac{H_S}{H_S + \kappa_{CH}} C \tag{12}$$

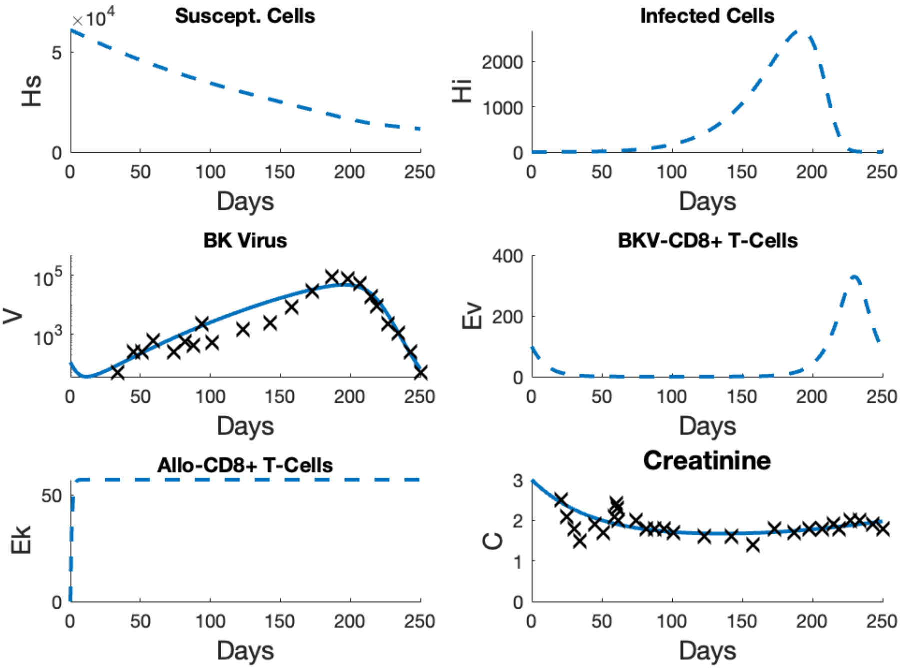

Figure S4. Model 1 fit to EHR patient #4947 data without characteristic functions. The model fit captures the data while V and E_V to get close to 0 since the characteristic functions are removed.

Given the less than desired outcomes, further adjustments to the model are necessary. The second model improvement pertains to the constant source terms, λ_{EV} and λ_{EK} in the equations of the effector cells, (10) and (11) respectively. These terms are part of the original model in [1] and regarded as small source terms. The constant λ_{EV} had no explanation. Similarly, λ_{EK} was intended to be related to HLA matching also without explanation, but HLA matching can be accounted for with the maximal growth rate ρ_{EK} in the allo-specific effector cell equation, (11). Removing λ_{EV} and λ_{EK} is plausible since they are not biologically necessary for the model.

Examining the sensitivity analysis in Figure 3ab, the viral state is sensitive to λ_{EV} and only locally toλ_{EV} but not ρ_{EK} and ρ_{EV} (the growth rates based on the magnitude of the threat). This indicates that λ_{EV} and λ_{EK} are driving the growth of the CD8+ T cell populations. In our view, it is more important that the threat dependent growth rates of the effector cells are the factors driving the immune response while also being easier to estimate. Hence, λ_{EK} and λ_{EV} are removed from the model in order to make the viral state sensitive to ρ_{EK} and ρ_{EV}. In Figure 3d, the viral state is sensitive to ρ_{EV} but not ρ_{EK}. Although, ρ_{EK} is the only growth rate in Equation (11) and estimation is necessary to adjust the allo-specific immune response based on patient data. Thus both parameters are estimated, see Table S1. This adjustment provides the benefit of reducing the size of the parameter space and also can be observed in model 1.1 Equations (16) and (17).

Immune Response Model: Model 1.1

$$\dot{H_S} = -\beta H_S V - \tilde{\beta} H_S E_K \tag{13}$$
$$\dot{H_I} = \beta H_S V - \delta_{HI} H_I - \delta_{EH} E_V H_I \tag{14}$$
$$\dot{V} = \rho_V \delta_{HI} H_I - \delta_V V - \beta H_S V \tag{15}$$
$$\dot{E_V} = (1-\epsilon)\left(\frac{\rho_{EV} V}{V + \kappa_V} E_V\right) - \delta_{E_V} E_V \tag{16}$$
$$\dot{E_K} = (1-\epsilon)\left(\frac{\rho_{EK} H_S}{H_S + \kappa_{KH}} E_K\right) - \delta_{E_K} E_K \tag{17}$$
$$\dot{C} = \lambda_C - \delta_{C0} \frac{H_S}{H_S + \kappa_{CH}} C \tag{18}$$

When first fitting model 1.1 to the data from patient #4947 (solid blue trajectory in Figures S5a and S5b), the dynamics attempt to capture the trend of the BKPyV data, and the desired improvements in states H_S and E_K are recognized: H_S was stable with reasonable allograft loss and E_K did not immediately jump from 0 to a carrying capacity. Since the BKPyV dynamics do not fit the data as well as previous models, another model change is necessary. Of the estimated parameter values, there is over a magnitude of difference between the values of the natural loss rates of the effector cells, δ_{EV} and δ_{EK}. This leads to the final model change.

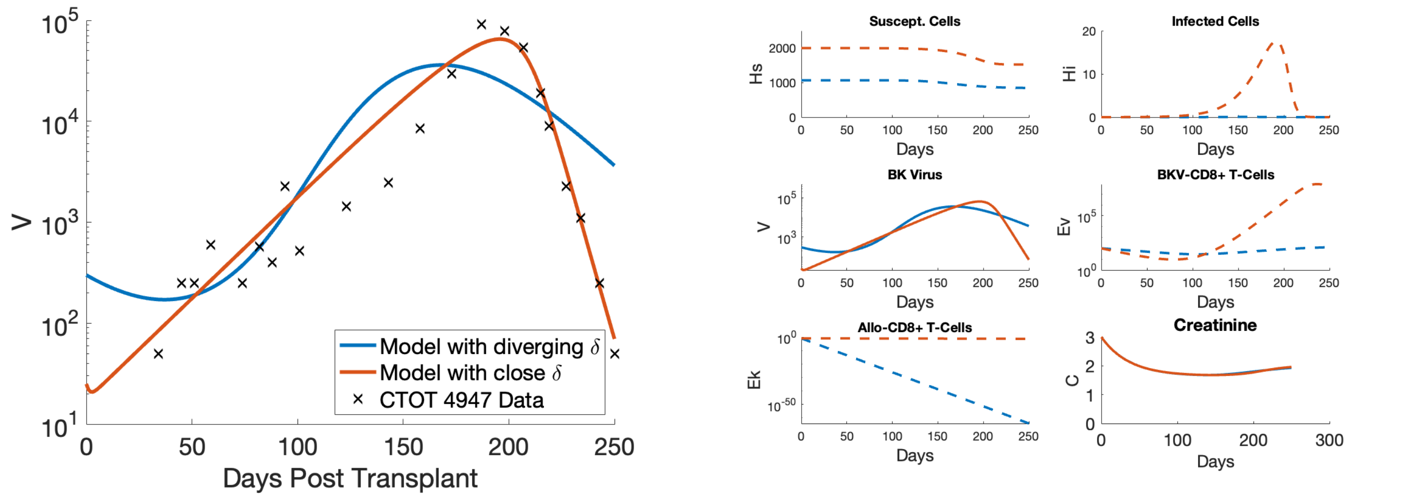

(a) BKPyV **(b)** All states

Figure S5. Model 1.1 fit to EHR patient #4947 BKPyV data with no linear source terms in effector cell equations. a) Restricting δ_{EV} and δ_{EK} to similar magnitudes (red trajectory) fit the patient data better than allowing these parameters to differ by 1 or more orders of magnitude (blue trajectory). b) The other model states show that H_S (top left panel) plateaus as desired for both trajectories while H_I (top right panel) and E_V(middle right panel) react more realistically when the delta parameters are restricted.

Just as model 1.1 saw changes in the effector cell dynamics, the change in δ's also occurs in Equations (16) and (17). model 1.1 assumes that the BKPyV-specific and the allo-specific cells have different death rates δ_{EV} and δ_{EK} respectively. Specific T cells originate from naive T cells indicating that specific T cells are similar in structure. Following other mathematical modeling of T cells, we adopt the assumption that all T cells have identical properties other than the pathogens they are specific to [6–9]. Furthermore it would be unlikely that the natural death rate for two unique specific T cells would differ by orders of magnitude. Instead we assume they lie within a relatively small range [10].

The red trajectory in Figure S5 is the result of estimating the parameters of model 1.1 where δ_{EV} and δ_{EK} are constrained to within an order of magnitude of each other. The

red trajectory displays a better fit to the BKPyV data addressing the concern of the blue trajectory. In the top right panel of Figure S5b, the infected cells increase with the BKPyV infection as expected and BKPyV-specific CD8+ T cells demostrate a stronger response. All indications of a more biologically realistic model solution.

For the updated immunosuppression model in the interest of simplicity, we assume that the death rates, δ_{EV} and δ_{EK}, are equal and therefore we label the joint death rate as δ_{EJ} in both Equations (22) and (23). This assumption does not apply to the proliferation rate of these equations because CD8+ T cell response to pathogens occurs in relation to the particular threat [6]. We assume the growth rates are unique to the BKPyV and allograft responses. This change to a joint death rate for effector cells further simplifies model and again reduces the parameter space. The Updated Immune Response Model (model 2) is described in the Equations (19) - (24) and the model flow chart, Figure S6.

Updated Immune Response Model: Model 2

$$\dot{H_S} = -\beta H_S V - \tilde{\beta} H_S E_K \tag{19}$$

$$\dot{H_I} = \beta H_S V - \delta_{HI} H_I - \delta_{EH} E_V H_I \tag{20}$$

$$\dot{V} = \rho_V \delta_{HI} H_I - \delta_V V - \beta H_S V \tag{21}$$

$$\dot{E_V} = (1-\epsilon)\left(\frac{\rho_{EV} V}{V + \kappa_V} E_V\right) - \delta_{EJ} E_V \tag{22}$$

$$\dot{E_K} = (1-\epsilon)\left(\frac{\rho_{EK} H_S}{H_S + \kappa_{KH}} E_K\right) - \delta_{EJ} E_K \tag{23}$$

$$\dot{C} = \lambda_C - \delta_{C0} \frac{H_S}{H_S + \kappa_{CH}} C \tag{24}$$

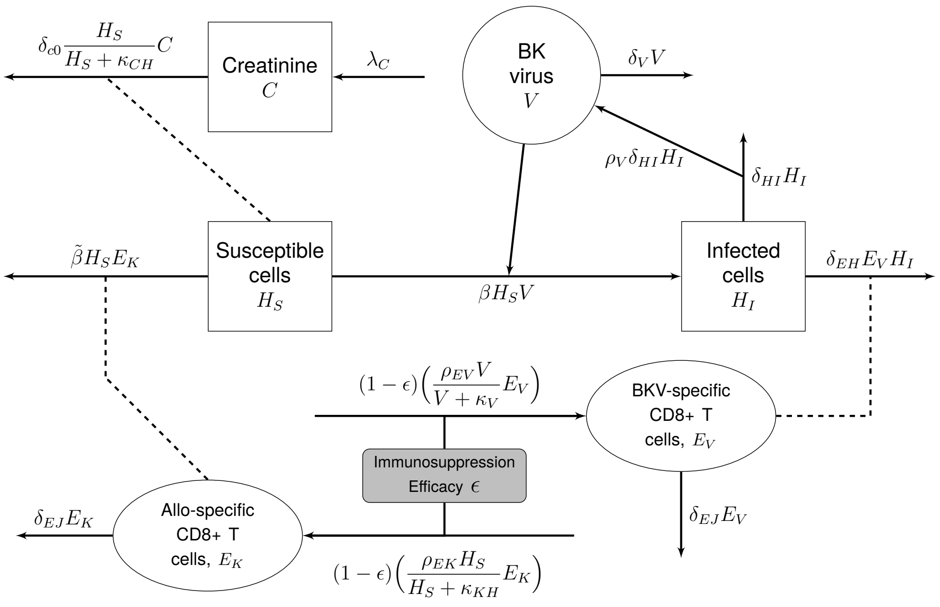

Figure S6. Diagram of the model 2 state interactions

We have refined and simplified this model into the Updated Immune Response Model (model 2). This model contains no characteristic functions or linear source terms for immune cells. We have also combined the natural death rate of the effector cells since it is unlikely two types of CD8+ T cells would die at significantly different rates. With these modifications,

the Banks et. al. immune response model has been reduced to 14 parameters from 20, not including the immunusuppression parameter ϵ. The updated model is verified qualitatively by gathering parameters from literature similar to model 0 [1,3] and also by estimating a subset of the parameters which the viral and creatinine states have sensitivity to. The parameter descriptions and default values are found in Table 2 in the section 2.

S3. Sensitivity Analysis

Morris screening is a one-at-a-time method which means that to calculate an elementary effect only one parameter value will be changed while all others remained fixed. Finite differences can be used in the calculation of elementary effects by using model states calculated with both the original sample parameters and the new perturbed sample parameter. For example, suppose the parameter space is 3-dimensional with sample vector (q_1, q_2, q_3). A perturbation, Δ, will be applied to each element in a random order (that changes for each sample vector). For example, (q_1, q_2, q_3) is the original sample and q_2 will be perturbed yielding sample $(q_1, q_2 + \Delta, q_3)$. Consider that your model output is described by $y(q)$, then the elementary effect for parameter q_i is

$$\text{Elementary Effect i} = \frac{y(q_1, \dots q_i + \Delta \dots, q_n) - y(q_1, \dots q_i \dots, q_n)}{\Delta} \tag{25}$$

where $i = 1, \dots, n$ is the parameter index and n is the number of parameters. All of the elementary effects for parameter i will be averaged together to obtain the ranking value. For a deeper explanation of the Morris screening method, see [11,12].

Our application of Morris screening to the immunosuppression model used a sample set of 10,000 points and $\Delta = 0.5556$ following from the formula in [11]. Our sample set was generated using the random number generator in Matlab with seed 50. The results of the initial screening for state BKPyV in the original model are in Figure 3a where we see that the subset of parameters including κ_V, $\tilde{\beta}$, λ_{EK}, δ_{EK}, λ_C, δ_{C0}, κ_{CH}, ρ_{EK}, κ_{KH}, and ρ_{EV} have little to no effect on the BK virus state. Hence, the remaining 8 sensitive parameters should be considered for estimation using real patient data.

For local sensitivity analysis, a derivative-based method is used which calculates partial derivatives at a point in the parameter space which we will call q^*, the nominal values of q. Consider our general set of parameters to be the vector q with $j = 1, \dots, 18$ different parameters and our state space as $x(q)$ with $i = 1, \dots, 6$ states. The partial derivatives $\partial x_i / \partial q_j$ represent the local sensitivities of the states with respect to the parameters as the change in the model state x_i over the small change in the parameter q_j. This method is inherently local since the partial derivatives are evaluated at q^*.

The sensitivity coefficients, as defined in [13], are derived from the partial derivatives and used as a metric for comparison between sensitivities of a state with respect to each parameter. The parameter spaces for different parameters can vary by many orders of magnitude and the calculation of the sensitivity coefficient normalizes each sensitivity. The sensitivity coefficients can be calculated using the formula:

$$S_i(q) = \left[\frac{1}{t_f - t_0} \int_{t_0}^{t_f} \left| \frac{\partial y}{\partial q_i} \frac{q_j}{\max_t y} \right|^2 dt \right]^{1/2} \tag{26}$$

where t_0 and t_f are the initial and final times respectively over which the model is run and $\max_t y$ is the maximum value the state of our model takes on this time interval. The sensitivity, $\partial y(t)/\partial q_i$, is calculated using the central difference formula with a step size $h_i = \sqrt{\text{Relative Tolerance}} * q_i$ [14]. Integration in time is solved numerically with the composite Simpson's rule. The results of this method are shown in Figure 3bd.

S4. Identifiability

To determine the identifiability of the sensitive parameters, eigenvalues of the sensitivity matrix are calculated. The sensitivity matrix consists of the partial derivatives found in the local sensitivity analysis arranged in a matrix as follows:

$$\tilde{S_{ij}} = \frac{\partial y}{\partial q_j}(t_i, q^*)\frac{q_j^*}{y(t_i, q^*)}. \tag{27}$$

We note that small eigenvalues of the $\tilde{S^T}\tilde{S}$ are problematic for identifying parameters so a small cutoff value of $e = 10^{-4}$ is used following [13,15]. This algorithm determines the set of identifiable parameters given e:

1. Compute the eigenvalues of $\tilde{S^T}\tilde{S}$ and order them:

$$|\lambda_1| \leq |\lambda_2| \leq \cdots \leq |\lambda_n|. \tag{28}$$

2. If $|\lambda_1| \leq e$, conclude this parameter is not identifiable, otherwise exit the algorithm.
3. Examine eigenvector v_1 for the largest component, E, which corresponds to the parameter that is least identifiable.
4. Remove the column of $\tilde{S}$ that corresponds to the position of E in v_1. Return to step 1 and repeat.

At the conclusion of the algorithm, the q_i's that correspond to the remaining eigenvalues consist of the set of identifiable parameters.

S5. Standard Errors

Standard errors and subset selection are determined using the Fisher information matrix and following the procedure demonstrated in [16]. Subset selection is implemented using the algorithm developed in [17], [18]. Figures S7 and S8 show the results of the subset selection in terms of normalized standard errors for the patients #4947, #287779, #287915, and #287660. The red threshold in the figures signal when the standard error matches the value of the parameter. Standard errors below the threshold are considered reasonable because the error is less than parameter value indicating a level of confidence in the parameter value. Parameters with standard errors above the threshold indicate there is not enough information present in the data to be confident in the estimated value of parameter or that the parameter is correlated with another. A way to interpret each figure is to focus on each column of standard errors where those reported are for the best set of parameter estimates for the group size indicated on the independent axis. From this point of view, the parameters in the set can be split between those above and below the threshold.

Two primary takeaways from these results are shown in the Figures S7 and S8 collectively. The first relates to obtaining the most parameter estimates with reasonable standard errors. For each patient estimating only three of the model parameters together can yield reasonable standard error for all three of them. Any other number of estimated parameters together produces at best two estimates with reasonable standard errors. The second takeaway stems from estimating eight parameters (final column in the figures) where seven or eight of the parameters have unreasonable standard errors. This result suggests that the model parameters are highly correlated with each other. This is not uncommon in nonlinear differential equations. Our goal in this work is not to determine nominal values (estimates with low standard error) of all of parameters in the model but rather produce acceptable model fits to patient data which the model achieves.

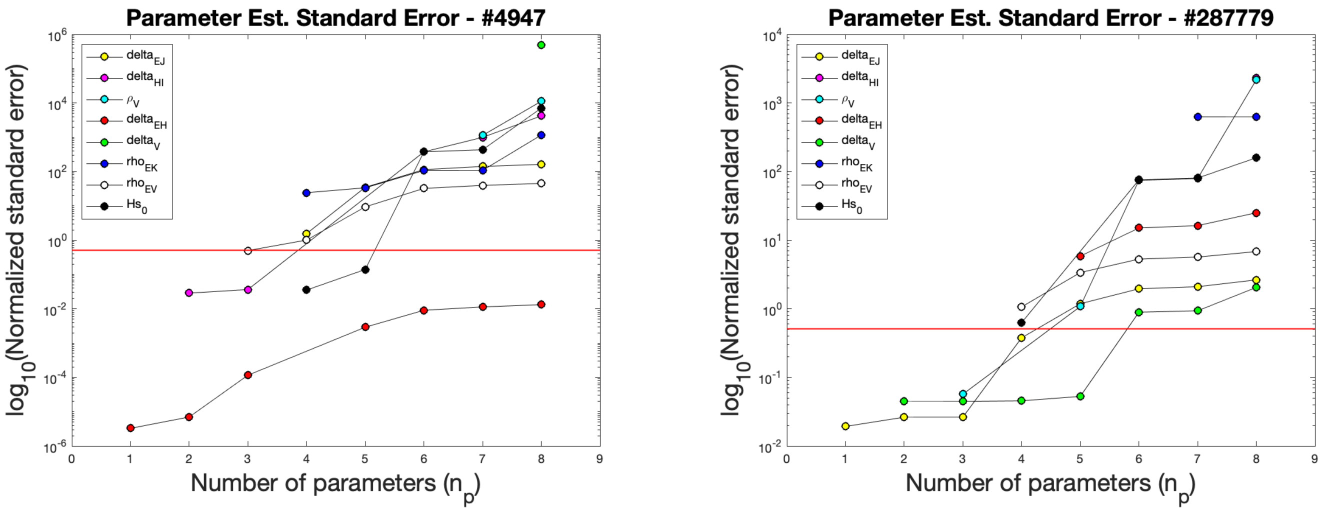

(a) Patient #4947

(b) Patient #287779

Figure S7. Parameter estimate subset selection based on standard errors for a) #4947 and b) #287779. Each number of parameters indicates the best standard errors for a subgroup of the size. The red line indicates where standard errors are greater then their respective parameter values. For patient #4947, at best two parameters can be estimated with acceptable standard errors, while for patient #287779 three parameters fit that criteria. Estimating all eight parameters as show in Table ?? shows that almost all estimates have high standard error and are likely correlated for both patients.

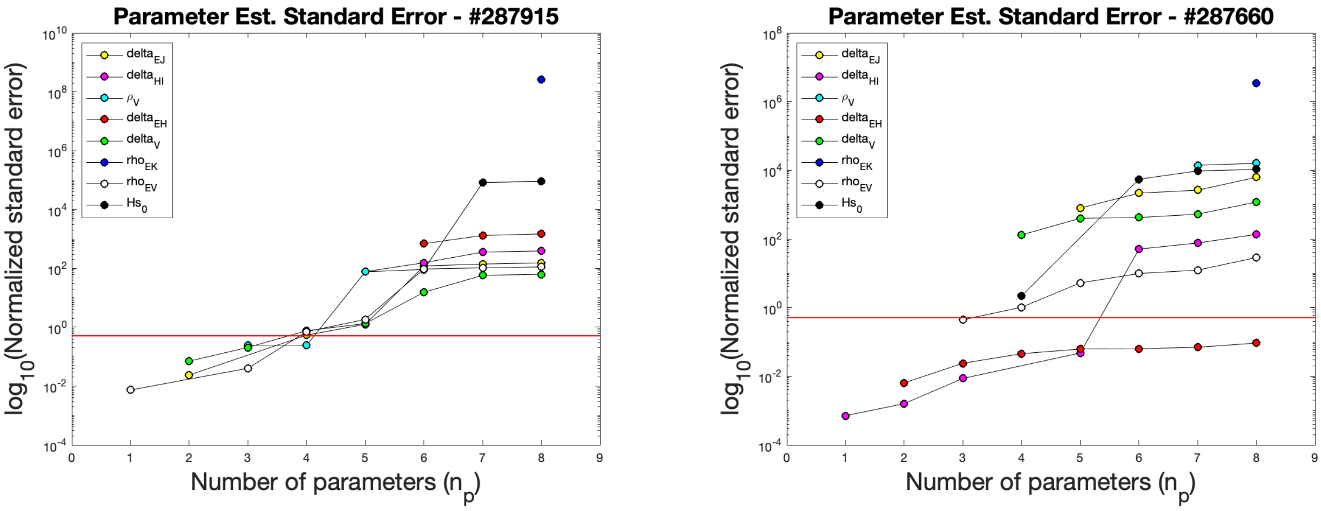

(a) Patient #287915

(b) Patient #287660

Figure S8. Parameter estimate subset selection based on standarad errors for a) #287915 and b) #287660. Each number of parameters indicates the best standard errors for a subgroup of the size. The red line indicates where standard errors are greater then their respective parameter values. For patient #287915, three parameters can be estimated with acceptable standard errors, while for patient #287660 only two parameters fit that criteria. Estimating all eight parameters as show in Table ?? shows that almost all estimates have high standard error and are likely correlated for both patients.

S6. Stability Analysis

S6.1. Equilibria

For exploring the general long term dynamics of an ODE model, stability analysis is a useful tool that couples these dynamics to model equilibria. Equilibria of the model can be determined by examining the system of equations that describe the model and utilizing some assumptions to separate them into cases. Each equilibrium of model can be described as attracting, repelling, or as a saddle point which is related to the long term model behavior near equilibrium. An equilibrium is a point in the solution space where given the model inputs there will be no change in the model output and the model output will remain at

that point for the remainder of the simulation. An equilibrium that is attracting finds that over time all model solutions near the equilibrium will approach and achieve the equilibrium. When the equilibrium is repelling, any model solution that is not the equilibrium as the starting point will move away from the equilibrium and the model output will never achieve the equilibrium. Finally, a saddle point is an equilibrium where some solutions attract to and achieve the equilibrium while other solutions actively move away from the equilibrium.

Updated Immune Response Model: Model 2

$$\dot{H_S} = -\beta H_S V - \tilde{\beta} H_S E_K \tag{29}$$

$$\dot{H_I} = \beta H_S V - \delta_{HI} H_I - \delta_{EH} E_V H_I \tag{30}$$

$$\dot{V} = \rho_V \delta_{HI} H_I - \delta_V V - \beta H_S V \tag{31}$$

$$\dot{E_V} = (1-\epsilon)\left(\frac{\rho_{EV} V}{V+\kappa_V} E_V\right) - \delta_{EJ} E_V \tag{32}$$

$$\dot{E_K} = (1-\epsilon)\left(\frac{\rho_{EK} H_S}{H_S+\kappa_{KH}} E_K\right) - \delta_{EJ} E_K \tag{33}$$

$$\dot{C} = \lambda_C - \delta_{C0} \frac{H_S}{H_S+\kappa_{CH}} C \tag{34}$$

The first step in stability analysis is determining the equilibria of the model. For reference, model 2 has been included in this section. An equilibrium is a point where the model solution is constant and the solution will remain at the equilibrium for all time in the future. This is true for model solutions that begin at the equilibrium and are also attracted to the equilibrium. A constant solution can be described mathematically by setting each model equation, (29) - (34), equal to zero representing that there are no changes in the state of that equation. For example, making Equation (29) constant requires setting the equation to zero as shown here:

$$0 = -\beta H_S V - \tilde{\beta} H_S E_K. \tag{35}$$

There are a couple core assumptions from the design of the model that influence the process of finding the equilibria after setting the ODE's equal to zero. The first model assumption states that all model parameters are greater than zero in all cases since the use of addition and subtraction in the model are all based on positive parameter values. In addition, efficacy is regarded as a fixed, positive parameter. The second assumption states all state variables (e.g., H_S, H_I, etc.) must be non-negative since negative quantities are not reasonable biologically. For example, there cannot be negative amounts of susceptible and infected renal cells. Since these are true to the model, they will be true and combined with other assumptions when determining the equilibria.

Core Assumptions for Stability Analysis:
1) All model parameters are > 0
2) All model variables are non-negative

S6.2. Case 1: There is No Trivial Solution

Now for the first case, we assume that $H_S = 0$ which is not terribly interesting since it represents the allograft being completely non-functioning. This choice is based on the fact that setting H_S to zero immediately sets $\dot{H_S}$ to zero, see Equation (36). Sub- stituting zero in for each H_S term in the system of equations results in the following changes.

Model 2 - Assuming $H_S = 0$

$$\dot{H_S} = 0 \tag{36}$$
$$\dot{H_I} = 0 - \delta_{HI}H_I - \delta_{EH}E_V H_I = 0 \tag{37}$$
$$\dot{V} = \rho_V \delta_{HI}H_I - \delta_V V - 0 = 0 \tag{38}$$
$$\dot{E_V} = (1-\epsilon)\left(\frac{\rho_{EV}V}{V+\kappa_V}E_V\right) - \delta_{EJ}E_V = 0 \tag{39}$$
$$\dot{E_K} = 0 - \delta_{EJ}E_K = 0 \tag{40}$$
$$\dot{C} = \lambda_C - 0 = 0. \tag{41}$$

Examining Equation (40), it is clear that E_K must be zero for $\dot{E_K} = 0$. Thus we have that $H_S = E_K = 0$ based on our assumptions. In Equation (37), setting $H_I = 0$ is the only way to not violate core assumption # 1. Since $H_I = 0$ now, Equation (38) will change so that V must also be 0. Substituting $V = 0$ into Equation (39) now forces $E_V = 0$. Hence we have that $H_S = H_I = V = E_V = E_K = 0$. The final equation, (41), indicates that there is no trivial equilibrium of the model. Instead this analysis indicates that if all susceptible renal cells are lost ($H_S = 0$) then the remaining states will also go to zero while state C continues constant growth of creatinine in the blood stream which is realistic biologically.

Case 1 Assumptions:
1) $H_S = 0$
2) $H_I = 0$
Result: There is no trivial equilibrium.

S6.3. Case 2: Biologically Reasonable (BR) Equilibrium

For the second case, we consider Equation (35) again. In case 1, H_S was assumed to be zero but for case 2 the initial assumption is that $V = E_K = 0$ to satisfy (35) which implies there are infinitely many possible static values for H_S that also satisfy this equation. The static value for the equilibrium will be designated $\bar{H}_S$. We denote the state values that satisfy the constant equations in bar notation, e.g., for state H_S the value of $\bar{H}_S$ satisfies the constant equations and is the value in the equilibrium. Substituting these values into model 2 produces the following constant equations for finding the equilibrium.

Model 2: Assuming $V = E_K = 0$

$$\dot{H_S} = -\beta\bar{H}_S * 0 - \tilde{\beta}\bar{H}_S * 0 = 0 \tag{42}$$
$$\dot{H_I} = -\delta_{HI}H_I - \delta_{EH}E_V H_I = 0 \tag{43}$$
$$\dot{V} = \rho_V \delta_{HI}H_I = 0 \tag{44}$$
$$\dot{E_V} = -\delta_{EJ}E_V = 0 \tag{45}$$
$$\dot{E_K} = 0 \tag{46}$$
$$\dot{C} = \lambda_C - \delta_{C0}\frac{\bar{H}_S}{\bar{H}_S + \kappa_{CH}}C = 0 \tag{47}$$

Now given this initial assumption, $E_K = 0$ indicates that $\dot{E}_K$ is constant as shown in (46). Next the assumption implies that $E_V = 0$ for Equation (45) to be true. Now with $E_V = 0$ then $H_I = 0$ given Equations (43) and (44). To conclude the process, the constant equation

for C, (47), can be simplified so that the equilibrium state $\bar{C}$ can be described as a function of the steady state value $\bar{H}_S$ as shown here,

$$\bar{C}(\bar{H}_S) = \frac{\lambda_C(\bar{H}_S + \kappa_{CH})}{\delta_{C0}\bar{H}_S}. \tag{48}$$

Case 2 Assumptions:
1) $V = 0$
2) $E_K = 0$
Result: BR equilibrium has the form $(\bar{H}_S, 0, 0, 0, 0, \bar{C})$.

S6.4. Case 3: Biologically Unrealistic (BU) Equilibrium

To find the second equilibrium of the model, we start with Equation (35) again but relax core assumption 2 therefore model states can be negative. The reason for this is obvious when simplifying (35) to the form:

$$E_K = -\frac{\beta}{\tilde{\beta}}V \tag{49}$$

From Equation (49), it is clear that both V and E_K cannot be positive while core assumption 1 (model parameters are positive) is maintained. Since by design all parameters must be positive, this assumption is unalterable. Given that case 2 already addressed when V and E_K are both zero, case 3 focuses only on non-zero values of V and E_K. Ultimately, the result will be an equilibrium solution with a negative valued state $\bar{V}$ or $\bar{E}_K$ which creates a biologically unreasonable solution.

By setting the model equations to zero to find the equilibrium, simplifying the model equations in the following order(32),(33),(29), (31),(30),(34) generates the following equilibrium solution (given that E_V and E_K do not equal zero),

$$\bar{V} = \frac{\kappa_V\delta_{EJ}}{\rho_{EV}(1-\epsilon) - \delta_{EJ}} \tag{50}$$

$$\bar{H}_S = \frac{\kappa_{KH}\delta_{EJ}}{\rho_{EK}(1-\epsilon) - \delta_{EJ}} \tag{51}$$

$$\bar{E}_K = -\frac{\beta}{\tilde{\beta}}\bar{V} \tag{52}$$

$$\bar{H}_I = \frac{\delta_V + \beta\bar{H}_S}{\rho_V\delta_{HI}}\bar{V} \tag{53}$$

$$\bar{E}_V = \frac{\beta\bar{H}_S\bar{V}}{\delta_{EH}\bar{H}_I} - \frac{\delta_{HI}}{\delta_{EH}} \tag{54}$$

$$\bar{C} = \frac{\lambda_C(\bar{H}_S + \kappa_{CH})}{\delta_{C0}\bar{H}_S}. \tag{55}$$

Case 3 Assumptions:
1) Model States can be negative.
2) $V \neq 0$ **and** $E_K \neq 0$ **for Equation (35)**
3) $H_S \neq 0$, $E_V \neq 0$, **and** $E_K \neq 0$ **for division in (35), (32), and (33)**
4) $H_I \neq 0$ **for substitution and simplification**
Result: BU equilibrium has the form $(\bar{H}_S, \bar{H}_I, \bar{V}, \bar{E}_V, \bar{E}_K, \bar{C})$.

S6.5. Stability Dynamics

There are 2 equilibria for this model. One is biologically unrealistic while the other falls in the biologically reasonable solution space, i.e., all model states are non-negative. To examine their long term dynamics, a local linear stability analysis is performed to determine if long term outcomes will attract to or repel from the given equilibrium. Evaluating the Jacobian of the model is the first s tep i n l ocal l inear s tability a nalysis. T he v alues for the Jacobian matrix elements are calculated by using the equilibrium state values and the parameter values associated with determining that equilibrium. The next step in the linear stability analysis is to examine the eigenvalues for the Jacobian matrices which are shown in Table S2. Since a Jacobian matrix can be calculated for each equilibrium, there are two sets of eigenvalues to observe.

Table S2. Eigenvalues and equilibria: CTOT patient #287660

	BR Eigenvalues	BU Eigenvalues		BR Equilibrium	BU Equilibrium
λ_1	-0.1933	-0.2284	$\bar{H}_S$	100	501.3
λ_2	-0.0306	-0.0095	$\bar{H}_I$	0	3.151
λ_3	-0.0223	0.0023	$\bar{V}$	0	3125.3
λ_4	0.0076	-0.0023	$\bar{E}_V$	0	28.90
λ_5	-0.0020	0.001 + 0.0214i	$\bar{E}_K$	0	-2.577
λ_6	0	0.001- 0.0214i	$\bar{C}$	5.05	1.047

Since both sets of eigenvalues have elements with positive real parts and others with negative real parts, these equilibria are classified as saddle points. Real positive eigenvalues indicate a repelling nature from the equilibrium while real negative eigenvalues indicate an attracting nature to the equilibrium. Saddle points are unstable since there exist solutions that repel from the equilibrium, but despite the instability there also exist solutions that attract to the equilibrium. Dominant eigenvalues can help predict the behavior of solutions along the eigenvectors over time [19].

Consider CTOT patient 660 and the dominant eigenvalue associated with the BU equilibrium. The dominant eigenvalue, -0.2284, far exceeds the only positive eigenvalue of 0.0023 indicating a strong attraction to the BU equilibrium. Since the dominant eigenvalue is much larger, many model solutions will attract towards this equilibrium quickly in the direction of the dominant eigenvector. The two complex eigenvalues with very small positive real parts indicate that the solution will never achieve the equilibrium point. Instead solutions will approach the equilibrium and then oscillate around it. The only exception is if the initial state of the model solution is on an eigenvector associated with a real valued eigenvalue, then it will attract to or repel from the equilibrium based on the sign of the eigenvalue. Since this is a local analysis, the extent of the attraction to this saddle points is unknown. The elements in the BU equilibrium indicate that the BR and BU equilibria are far apart. Hence it is unclear if the attraction of BU equilibrium may attract some trajectories of solutions in the biologically reasonable space and prevent them from attracting to the BR equilibrium.

In the case of the BR equilibrium for patient 660, -0.1933 is the dominant eigenvalue while 0.0076 is the only positive eigenvalue. These values are not close in magnitude to each other indicates a strong attraction in the direction of the dominant eigenvector. If we allowed the model to run to infinite time eventually most solutions sufficiently close to the BR equilibrium will attract to it. The repelling quality of the second dominant eigenvalue may play a part in preventing some of the model solutions from getting close enough to attract to the BR equilibrium. The BR equilibrium that these eigenvalues are drawn from is $(100, 0, 0, 0, 0, 5.05)$ and could represent a transplant patient that fought off a BKPyV

infection which follows from the fact that H_S and V need to drop to very low values to eliminate E_V and E_K. Here $\bar{H}_S$ is so low that nephropathy would have occurred and the allograft would not be functioning. Hence this is not an equilibrium we want the model to approach.

Furthermore, the BR equilibrium is a relation between the two states $\bar{H}_S$ and $\bar{C}$ and it's value can shift based on the initial value of all model states. For instance, if we assumed a healthy individual without foreign kidney tissue, i.e., $H_I = V = E_V = E_K = 0$, then the BR equilibrium would be based on the initial volume of healthy kidney cells H_{S0}. An example would be $(5000, 0, 0, 0, 0.15)$ which has a dominant eigenvalue of -0.3836 and a second most dominant eigenvalue of 0.1975 which are somewhat close in magnitude. Here the dominant eigenvalue would attract model solutions while the second dominant would repel them a significant amount. This requires model solutions to be close to this equilibrium in order to attract to it. Realistically this equilibrium would be incredibly unlikely for a renal transplant patient with BKPyV reactivation to achieve. The use of a control theory framework would help guide model solutions toward a BR equilibrium with a high $\bar{H}_S$ value of 5000 or more, but would most likely fall short where $C > \bar{C}$.

In the following tables of eigenvalues and equilibria for other patients, similar conclusions can be drawn to those above.

Table S3. Eigenvalues and equilibria: CTOT patient #287915

	**BR Eigenvalues**	**BU Eigenvalues**		**BR Equilibrium**	**BU Equilibrium**
λ_1	-0.1828	-0.2284	$\bar{H}_S$	100	501.3
λ_2	-0.1064	-0.0095	$\bar{H}_I$	0	3.151
λ_3	-0.0936	0.0023	$\bar{V}$	0	3125.3
λ_4	0.0124	-0.0023	$\bar{E}_V$	0	28.90
λ_5	-0.0020	0.001 + 0.0214i	$\bar{E}_K$	0	-2.577
λ_6	0	0.001- 0.0214i	$\bar{C}$	5.05	1.047

Table S4. Eigenvalues and equilibria: CTOT patient #287779

	**BR Eigenvalues**	**BU Eigenvalues**		**BR Equilibrium**	**BU Equilibrium**
λ_1	-0.0748	-0.5984	$\bar{H}_S$	100	-7494.99
λ_2	-0.0689	0.3715	$\bar{H}_I$	0	2.916
λ_3	-0.0292	-0.0015 + 0.0331i	$\bar{V}$	0	1858.85
λ_4	0.0043	-0.0015 - 0.0331i	$\bar{E}_V$	0	-5.69
λ_5	-0.0020	0 + 0.0013i	$\bar{E}_K$	0	-1.53
λ_6	0	0- 0.0013i	$\bar{C}$	5.05	-0.0167

Table S5. Eigenvalues and equilibria: EHR patient #4080

	**BR Eigenvalues**	**BU Eigenvalues**		**BR Equilibrium**	**BU Equilibrium**
λ_1	-0.0422	0.0396	$\bar{H}_S$	100	-1029.9
λ_2	-0.0421	0.0230	$\bar{H}_I$	0	1.445
λ_3	-0.0296	-0.0115 + 0.0279i	$\bar{V}$	0	724.6
λ_4	0.0036	-0.0115 - 0.0279i	$\bar{E}_V$	0	-1597.0
λ_5	-0.0020	0 + 0.0092i	$\bar{E}_K$	0	-0.5956
λ_6	0	0 - 0.0092i	$\bar{C}$	5.05	-0.4355

Table S6. Eigenvalues and equilibria: EHR patient #4947

	BR Eigenvalues	BU Eigenvalues		BR Equilibrium	BU Equilibrium
λ_1	-0.1447	-0.1506	$\bar{H}_S$	100	377.2
λ_2	-0.0300	-0.0073	$\bar{H}_I$	0	3.261
λ_3	-0.0200	-0.0011	$\bar{V}$	0	691.9
λ_4	-0.0020	0.0011	$\bar{E}_V$	0	1804.5
λ_5	0.0016	0.0005 + 0.012i	$\bar{E}_K$	0	-0.5687
λ_6	0	0.0005 - 0.012i	$\bar{C}$	5.05	1.376

References

1. Banks, H.T.; Hu, S.; Link, K.; Rosenberg, E.S.; Mitsuma, S.; Rosario, L. Modelling immune response to BK virus infection and donor kidney in renal transplant recipients. *Inverse problems in science and engineering* **2016**, *24*, 127–152.
2. Murad, N. Quantitative Modeling and Optimal Control of Immunosuppressant Treatment Dynamics in Renal Transplant Recipients. PhD thesis, North Carolina State University, 2018.
3. Murad, N.; Tran, H.; Banks, H. Optimal control of immunosuppressants in renal transplant recipients susceptible to BKV infection. *Optimal Control Applications and Methods* **2019**, *40*, 292–309.
4. Alcendor, D.J. BK polyomavirus virus glomerular tropism: implications for virus reactivation from latency and amplification during immunosuppression. *Journal of Clinical Medicine* **2019**, *8*, 1477.
5. Janeway, C.J.; P, T.; et. al., W.M. *Immunobiology: The Immune System in Health and Disease, 5th Ed.*; Garland Science, 2001.
6. Antia, R.; Ganusov, V.V.; Ahmed, R. The role of models in understanding CD8 + T-cell memory. *Nature reviews. Immunology* **2005**, *5*, 101–111.
7. Ribeiro, R.M.; Mohri, H.; Ho, D.D.; Perelson, A.S. In vivo Dynamics of T Cell Activation, Proliferation, and Death in HIV-1 Infection: Why Are CD4+but Not CD8+T Cells Depleted? *Proceedings of the National Academy of Sciences - PNAS* **2002**, *99*, 15572–15577.
8. Terry, E.; Marvel, J.; Arpin, C.; Gandrillon, O.; Crauste, F. Mathematical model of the primary CD8 T cell immune response: stability analysis of a nonlinear age-structured system. *Journal of Mathematical Biology* **2012**, *65*, 263–291. https://doi.org/https://doi.org/10.1007/s00285-011-0459-8.
9. Appay, V.; Rowland-Jones, S.L. Lessons from the study of T-cell differentiation in persistent human virus infection. *Seminars in Immunology* **2004**, *16*, 205–212. Anti-Viral Immunity, https://doi.org/https://doi.org/10.1016/j.smim.2004.02.007.
10. Thomas-Vaslin, V.; Altes, H.K.; de Boer, R.J.; Klatzmann, D. Comprehensive Assessment and Mathematical Modeling of T Cell Population Dynamics and Homeostasis1. *The Journal of Immunology* **2008**, *180*, 2240–2250.
11. Smith, R.C. *Uncertainty Quantification: Theory, Implementation, and Applications*; siam, 2014.
12. Campolongo, F.; Cariboni, J.; Saltelli, A. An effective screening design for sensitivity analysis of large models. *Environmental Modelling & Software* **2007**, *22*, 1509–1518. Modelling, computer-assisted simulations, and mapping of dangerous phenomena for hazard assessment, https://doi.org/https://doi.org/10.1016/j.envsoft.2006.10.004.
13. Arthur, J.G.; Tran, H.T.; Aston, P. Feasibility of parameter estimation in hepatitis C viral dynamics models. *Journal of Inverse and Ill-Posed Problems* **2017**, *25*, 69–80.
14. Dennis Jr, J.E.; Schnabel, R.B. *Numerical methods for unconstrained optimization and nonlinear equations*; SIAM, 1996.
15. Quaiser, T.; Mönnigmann, M. Systematic identifiability testing for unambiguous mechanistic modeling–application to JAK-STAT, MAP kinase, and NF-κ B signaling pathway models. *BMC systems biology* **2009**, *3*, 1–21.

16. Banks, H.T.; Beraldi, R.; Cross, K.; Flores, K.; McChesney, C.; Poag, L.; Thorpe, E. Uncertainty quantification in modeling HIV viral mechanics. *MBE* **2015**, *12*, 937–964. https://doi.org/https://doi-org/10.3934/mbe.2015.12.937.
17. Cintron-Arias, A.; Banks, H.; Capaldi, A.; Lloyd, A. A sensitivity matrix based methodology for inverse problem formulation. *J. Inverse and Ill-posed Problems* **2009**, *17*, 545–564.
18. Banks, H.T.; Cintron-Arias, A.; Kappel, F. Parameter selection methods in inverse problem formulation. *Mathematical Modeling and Validation in Physiology* **2013**, *2064*, 43–73. ser. Lecture Notes in Mathematics (eds. J.J. Batzel, M. Bachar, and F. Kappel), Springer Berlin Heidelberg.
19. Bohl, D.L.; Brennan, D.C. *Matrix Methods: Applied Linear Algebra and Sabermetrics, 4th Ed.*; Academic Press: Massachusetts, 2021.
